# Enigmatic mechanism of the *N*-vinylpyrrolidone hepatocarcinogenicity in the rat

**DOI:** 10.1007/s00204-021-03151-8

**Published:** 2021-09-30

**Authors:** Franz Oesch, Daniela Fruth, Jan G. Hengstler, Eric Fabian, Franz Ingo Berger, Robert Landsiedel

**Affiliations:** 1 Oesch-Tox Toxicological Consulting and Expert Opinions GmbH&CoKG, Rheinblick 21, 55263 Ingelheim, Germany; 2 Institute of Toxicology, Johannes Gutenberg University, 55131 Mainz, Germany; 3 Experimental Toxicology and Ecology, BASF SE, 67056 Ludwigshafen am Rhein, Germany; 4 Present Address: Knoell Germany GmbH, Eastsite XII, Konrad-Zuse-Ring 25, 68163 Mannheim, Germany; 5 Leibniz Research Centre for Working Environment and Human Factors (IfADo), University of Dortmund, Dortmund, Germany; 6 Regulatory Toxicology Chemicals, BASF SE, 67056 Ludwigshafen am Rhein, Germany

**Keywords:** N-vinylpyrrolidone, Genotoxicity, Carcinogenicity, Non-genotoxic carcinogen, Comet assay, Micronucleus

## Abstract

*N*-vinyl pyrrolidone (NVP) is produced up to several thousand tons per year as starting material for the production of polymers to be used in pharmaceutics, cosmetics and food technology. Upon inhalation NVP was carcinogenic in the rat, liver tumor formation is starting already at the rather low concentration of 5 ppm. Hence, differentiation whether NVP is a genotoxic carcinogen (presumed to generally have no dose threshold for the carcinogenic activity) or a non-genotoxic carcinogen (with a potentially definable threshold) is highly important. In the present study, therefore, the existing genotoxicity investigations on NVP (all showing consistently negative results) were extended and complemented with investigations on possible alternative mechanisms, which also all proved negative. All tests were performed in the same species (rat) using the same route of exposure (inhalation) and the same doses of NVP (5, 10 and 20 ppm) as had been used in the positive carcinogenicity test. Specifically, the tests included an ex vivo Comet assay (so far not available) and an ex vivo micronucleus test (in contrast to the already available micronucleus test in mice here in the same species and by the same route of application as in the bioassay which had shown the carcinogenicity), tests on oxidative stress (non-protein-bound sulfhydryls and glutathione recycling test), mechanisms mediated by hepatic receptors, the activation of which had been shown earlier to lead to carcinogenicity in some instances (Ah receptor, CAR, PXR, PPARα). No indications were obtained for any of the investigated mechanisms to be responsible for or to contribute to the observed carcinogenicity of NVP. The most important of these exclusions is genotoxicity. Thus, NVP can rightfully be regarded and treated as a non-genotoxic carcinogen and threshold approaches to the assessment of this chemical are supported. However, the mechanism underlying the carcinogenicity of NVP in rats remains unclear.

## Introduction

*N*-vinylpyrrolidone (NVP) (CAS-Nr. 88-12-0) is produced yearly up to several thousand tons to be used as monomer and mostly as starting molecule for producing polyvinyl pyrrolidone which in turn is widely used in pharmaceutics, cosmetics and food technology.

**Fig. 1:**
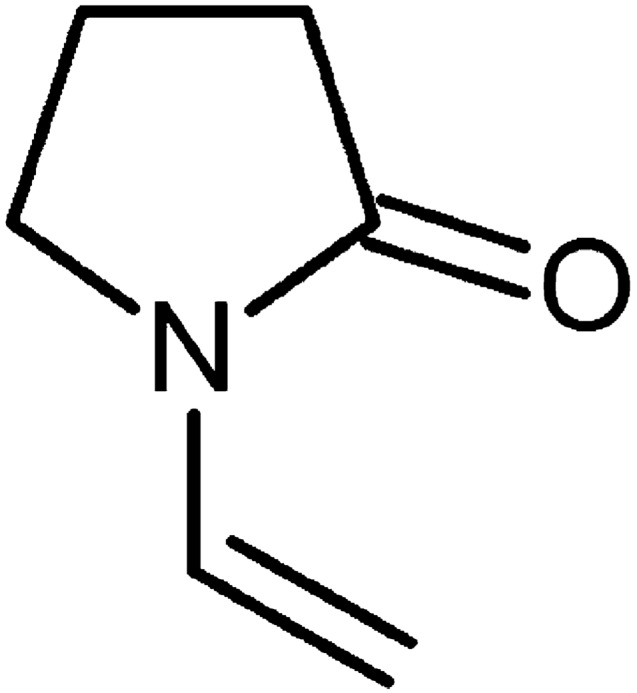
Structure of N-vinylpyrrolidone (NVP)

Previous studies showed toxicity of NVP especially to the target organs liver (increased gamma-glutamyl transpeptidase and glutathione: LOAEC in rats 5 ppm, in mice 10 ppm; foci of altered hepatocytes in rats at 5 ppm) and nasal mucosa (nasal cavity inflammation, olfactory epithelium atrophy as well as hyperplasia of the olfactory and respiratory epithelium basal cells: LOAEC in rats and mice 5 ppm). In addition, the number of erythrocytes, hematocrit and hemoglobin were reduced in rats and mice at 15 ppm (Klimisch et al. 1997b). Upon inhalation NVP was carcinogenic in the rat liver, nasal cavity and larynx (Table 1). The relative potency was highest for the liver, where hepatocellular carcinomas were observed already at the lowest tested dose, 5 ppm (Klimisch et al. 1997a). From the vinyl group of NVP (for NVP structure see Fig. 1) conceivably an electrophilically reactive epoxide could be formed as a metabolite, potentially leading to DNA damage, which might be regarded responsible for the observed carcinogenicity. This would be in line with the observed relative highest carcinogenic potency in the liver, an organ rich in xenobiotic oxidative metabolism (Oesch-Bartlomowicz and Oesch 2007). However, NVP was negative in a battery of genotoxicity tests, the Ames *Salmonella* back mutation test (three independent tests in four *Salmonella* strains), the *Klebsiella pneumoniae* fluctuation (point mutation) test, mammalian cell mutation tests (HGPRT and TK locus tests in L5178Y mouse lymphoma cells), the BALB/3T3 cell transformation test, the Unscheduled DNA Synthesis (UDS) test in rat hepatocytes as well as the chromosome aberration test in human lymphocyte cultures (MAK, 1994). In order to estimate from the observed carcinogenicity in the rat the carcinogenic risk for humans attempts to elucidate the mechanism of the carcinogenicity in the rat more clearly are highly desirable.

**Table 1:** Summary on the incidence of neoplasms in Sprague–Dawley rats treated by inhalation with *N*-vinyl-2-pyrrolidone for 24 months

Sex	Male				Female			
Exposure concentration (ppm)	0	5	10	20	0	5	10	20
Number of animals	70	60	60	60	70	60	60	60
Nasal cavity								
Adenoma	0	9**	9***	11***	0	2	8**	14***
Adenocarcinoma	0	0	4*	6*	0	0	0	4*
Liver								
Hepatocellular carcinoma	1	6*	5*	17***	1	3	6*	26***
Larynx Squamous carcinoma	0	0	0	4	0	0	0	4

Data from Klimisch et al. (1997a)

*P < 0.05

**P < 0.01

***P < 0.001

The following potential mechanisms for the carcinogenicity of NVP were tested, largely focusing on the rat liver, the most sensitive organ for the NVP carcinogenicity in the rat:

- In order to assess/even more completely exclude a genotoxic mechanism the above-mentioned already quite numerous genotoxicity tests were complemented by the following

- an in vivo micronucleus test in rat bone marrow and by

- a Comet assay in rat liver and lung cells in presence and absence of the DNA repair enzyme formamido-pyrimidine-DNA-glycosylase (FPG)

- Oxidative stress

- Non-protein-bound sulfhydrylsNon-protein-bound sulfhydryls

- Glutathione (GSH) recycling assay

- Receptor-mediated mechanisms

- Aryl hydrocarbon receptor (AhR)

- Constitutive androstane receptor (CAR)

- Pregnan X receptor (PXR)

- Peroxisome proliferator-activated receptor (PPAR)

- Histology of the rat liver

- Hematoxylin-Eosin

- Oil red

## Materials and methods

### Animals

The animal experiments described in this publication were approved by the responsible authority (Landesuntersuchungsamt Koblenz, Approval-No. G 13–3-042).

Eight-week-old male and female Wistar Crl:WI (Han) rats were obtained from Charles River Laboratories, Sulzfeld, Germany and acclimatized for 10 days. They were kept at 20–24 °C at a humidity of 30–70% in groups of six animals in polysulfone cages (Tecniplast) with free access to water and feed (10 mm pellets, Provimi Kliba, Kaiseraugst, Switzerland).

### Inhalation experiments

The animals were acclimatized to the whole-body exposure chambers for 2 days using the inhalation stream without the test substance. After this, rats were assigned randomly to the following test groups: Six rats of each gender and at each dose level were exposed to 0, 5, 10 or 20 ppm NVP for five consecutive days 6 h per day, all of this in analogy to the carcinogenicity bioassay (Klimisch et al. 1997a). As positive control rats were once treated orally with ethyl methanesulfonate [200 mg/kg body weight (bw) dissolved in saline (0.9% NaCl)] and killed 24 h later. Animals were weighed before the 2-day acclimatation to the exposure chambers and subsequently before and after the exposure to the test substance. Animals were killed in the morning after the last exposure period to perform the examinations described further down.

For the inhalation experiments, two animals per cage (DK III, Becker & CO., Castrop-Rauxel, Germany) were kept in the glass–steel inhalation chambers (volume approximately 200 L) without access to feed and water. For each test dose a constant amount of NVP was pumped by continuous infusion pumps PHD Ultra (Havard Apparatus, Holliston) to an evaporator (Glasverdampfer BASF, Ludwigshafen, Germany; Thermostat Julabo Labortechnik, Seelbach, Germany). The thus generated vapor was combined with air and introduced into the exposure chamber. This atmosphere then left the chamber via a reduced pressure exhaust system in order to ascertain avoidance of spurious contamination of the laboratory air potentially caused by a leakage of the system. The NVP concentration in the inhalation atmosphere was monitored by GC/FID (Agilent 6890 with autosampler, Dionex Chromeleon Software, column: ZB-WAXplus, Zebron, injection temperature 250 °C, flow rate 1 mL/min, carrier gas helium, FID detector, retention time about 4 min).

### Preparation of animal tissues

Animals were anesthetized by 3.5% isoflurane (approximately 3 min, evaporator Dräger Vapor 19.3) and then killed by cervical dislocation. The liver was removed and its left lateral lobe used for the Comet assay. The remainder of the liver was washed in saline and then in two aliquots shock frozen in liquid nitrogen and stored at − 80 °C for later examinations. Subsequently the lung was removed from the dead animal. Its left lobe was used for the Comet assay and the remainder treated as described above for the liver. Thereafter the femora were removed for the preparation of the bone marrow.

### Genotoxicity

#### Micronucleus test

##### Cellulose columns

13-mm filter disks with a pore size of 8 µm were placed on the bottom of 5-mL plastic syringes. Approximately 500 mg of a mixture consisting of equal parts microcrystalline cellulose (Sigmacell®, Typ 50) and alpha-cellulose fibers was added up to the 2-mL mark.

##### Bone marrow preparation

Bone marrow preparation was performed according to published methods (Romagna and Staniforth 1989; Salamone et al. 1980). The soft tissue was removed from the femora and the epiphyses were cut off. The bone marrow was rinsed off the diaphyses with approximately 3 mL 37 °C-warm fetal calf serum (FCS) (Biochrom, Berlin, Germany) per femur, placed on the above-described cellulose column and eluted with 3 mL of Hank's Balanced Salt Solution (HBSS) (Biochrom, Berlin, Germany) (with Ca^2+^ und Mg^2+^). The eluate was centrifuged at 300 g for 5 min and the pellet was suspended in 50 mL FCS. From this suspension three portions per femur were individually placed on slides, air-dried and then stained for 4 min in May-Grunwald solution 1 (Merck, Darmstadt, Germany), followed by 4 min in May–Grunwald solution 2 (May-Grunwald solution 1 diluted 1:1 with aqua bidest.), rinsed in aqua bidest and then stained with Giemsa solution (7.5% dilution derived from Giemsa obtained from Merck, Darmstadt, Germany) for 15 min. Subsequently, the slides were washed twice with aqua bidest, then dried, cleared in xylene and covered with Corbit-Balsam (I. Hecht, Kiel-Hasse, Germany).

##### Evaluation

2000 polychromatic erythrocytes (PCE) per animal (1000 per slide) were evaluated at 1000-fold magnification under immersion oil for the presence/absence of large and small micronuclei as well as the proportion of PCE among the total erythrocytes. Statistical significance of differences between treated *versus* untreated groups was evaluated by the one-sided Wilcoxon test (using the program MUKERN). The result is regarded as positive when the following criteria are fulfilled: 1. The number of PCE with micronuclei is statistically significantly and dose-dependently elevated; 2. the number of PCE with micronuclei exceeds the number in the present negative control group AND in the historical negative controls. The result is regarded as negative when the following criteria are fulfilled: The number of PCE with micronuclei is not statistically significantly greater than in the negative controls of the actual experiment AND lies within the historical negative control values.

#### Comet assay

The Comet assay was performed under alkaline conditions according to the principles described by Singh et al. (1988) and Tice et al. (2000).

##### Preparation of single cell suspensions

Liver and lung cells were obtained essentially as described by Hartmann et al. (2004) and by the Japanese Center for the Validation of Alternative Methods (JaCVAM 2009). Single cells were obtained within 1 h after killing of the animals in order to minimize DNA damage during the preparation.

The organ parts (see above) were washed in mincing buffer (50 mM EDTA in HBSS [without Ca^2+^ and Mg^2+^] pH 7.5: DMSO 9:1 (v/v), the latter added shortly before use, stored at 4 °C), cut into 1 to 2-mm pieces in ice-cold mincing buffer and transferred to a cell sieve with 70-µm pores. The viability of the cell suspension was evaluated by the Trypan Blue Assay (Strober 2001).

##### Preliminaries to the electrophoresis

100 µL of the obtained cell suspension was mixed at 37 °C with 900 µL Low Melting Agarose (LMA) (0.7% agarose in phosphate-buffered saline [PBS] [Biochrom, Berlin, Germany] without Ca^2+^ and Mg^2+^). 100 µL of this mixture was transferred to a slide, which had been coated with Normal Melting Agarose (NMA) (0.6% agarose in PBS without Ca^2+^ and Mg^2+^). The slides were protected with a cover slip and the agarose was hardened at approximately 4 °C. The cover slips were then removed and the slides put over night at 4 °C into lysis buffer (2.5 M NaCl, 125 mM NaOH, 127 mM EDTA, 10 mM Tris/NaOH, pH 10; 1% Triton X-100 and 10% DMSO (v/v), the latter two added before use) in order to lyse the cell membrane, nuclear membrane and remove the histone.

The then following procedures were performed in a shaded room to minimize UV-mediated DNA damage during the procedures.

Those slides which were destined to be treated with the DNA base excision repair enzyme FPG (Sigma Aldrich, Steinheim, Germany) were washed (3 times for 5 min) with FPG buffer (0.04 M HEPES, 0.5 mM EDTA, 0.1 M KCl, pH 8; add shortly before use bovine serum albumin [BSA] to a final concentration of 0.02%). Subsequently 50 µL FPG-enzyme-buffer (prepared shortly prior to use by 30-fold dilution of the following suspension in FPG buffer: 7 µL FPG-enzyme (14 U) and 70 µL glycerol in 623 µL FPG-Puffer, stored at − 80 °C) was pipetted on the slide which was covered with a cover slide and incubated for 30 min at 37 °C while being placed on a humid filter paper. After hardening of the agarose cover slips were removed.

Remaining lysis buffer or FPG buffer was removed by immersing/washing the slides in PBS (without Ca^2+^ and Mg^2+^).

##### Electrophoresis

The slides were then placed into the electrophoresis chamber, which was cooled and protected from light. Slides were covered with electrophoresis buffer (0.3 M NaOH, 1 mM EDTA, pH > 13) carefully avoiding air bubbles. The DNA was allowed to unwind (20 min). Thereafter the electrophoresis was started (25 V, 300 mA, 0.85 V/cm^2^, 30 min). The slides were then washed (2 times 5 min) in neutralization buffer (0.4 M Tris/HCl, pH 7.5), dehydrated and fixed in 100% ethanol (1 min), dried at room temperature overnight and stored at room temperature protected from dust.

##### Evaluation

Quantitative evaluation of the Comet assays was performed by fluorescence microscopy after DNA staining with ethidium bromide. 40 µL of a 0.0005% ethidium bromide solution was pipetted on the electrophoresed DNA carrying slide, which was then covered with a slip and immersion oil was added for microscopy. Counting and evaluation of DNA (comets-shaped and non-comet shaped) was performed semiautomatically using Comet Assay IVTM (Perceptive Instruments, Bury St Edmunds) at intensity grade 2 of the fluorescence microscope. 100 cells were counted per animal and per organ on two separate slides (50 per slide). Each of the two slides was evaluated by a separate person. The criteria of the Comet standardization atlas (Nakajima et al. 2009) were carefully followed such as avoiding the inclusion into the evaluation of cells on the border of the slide as well as hedgehogs (diffuse cells without a discernible head). Hedgehogs were counted separately.

As basis for the evaluation of the DNA damage the relative tail intensity (TI) was used, providing relative fluorescence intensity in the tail and thus information on the amount of DNA in the tail which is linearly related to the amount of DNA strand breaks. Therefore, this parameter is recommended for DNA damage evaluation within the Comet assay (Collins et al., 2008; Hartmann et al., 2003; Lovell & Omori, 2008). For obtaining the TI of the individual animal and organ the mean of the median TI per slide was calculated. These mean TI values per organ and animal were used to calculate the mean TI value per dose group.

A test substance is regarded as positive in the Comet assay, if the mean of the TI is dose-dependently increased and if the mean of the TI of a dose group is increased by a factor of 2 above the vehicle-only control group of the actual experiment. In difficult cases, if there is only a slight increase of the TI of some of the animals of the dose group, the decision is taken case by case. A statistical evaluation (two-sided *t*-test) was taken into consideration.

### Oxidative stress

#### Non-protein-bound sulfhydryl groups (NPSH)

Determination of NPSH was performed according to the principle described by Ellmann (1958, 1959) in the liver homogenate supernatant fraction of the NVP-exposed male Wistar rats. One g liver was homogenized at 0 °C in 9 mL of a 5% (weight/volume) aqueous 5-sulfosalicylic acid (5-SSA) solution, followed by centrifugation (14 000 g, 10 min, 4 °C). The supernatant fraction was stored in aliqots at − 80 °C. Prior to assay, the samples were diluted fourfold and to 100 µL of the diluted sample 200 µL Tris/EDTA (1 M/5 mM, pH 8.9) buffer was added. Determinations were performed in triplicates. As quality control 480 µM GSH was used, which was diluted by a factor of 5 for application in the assay. Reaction was started by addition of 5 µL of Ellmann’s reagent (10 mM 5,5′-dithiobis-2-nitrobenzoic acid [DTNB] in methanol, freshly prepared every day). Measurements were performed 5 min thereafter at *λ* = 412 nm.

#### Glutathione recycling assay

Glutathione recycling assay was adapted from the method described by Gallagher (1994) and performed using the 14 000 g supernatant fraction derived from the 5-SSA-treated liver homogenate of the NVP-exposed male Wistar rats as described above under “Non-protein-bound sulfhydryl groups”. The obtained samples were diluted fivefold with the 5% 5-SSA solution.

For determination of total glutathione (tGSH, i.e. GSH + GSSG) a GSH control (640 µM in 5% 5-SSA, diluted by a factor of 4 for application in the assay) and a tGSH control (200 µM in 5% 5-SSA, containing 10% GSSG) were treated the same way as the samples. To 5 µL of sample, controls as well as GSH standards (dissolved in 5% 5-SSA solution) 190 µL tGSH master mix, consisting of phosphate/EDTA buffer (125 mM KH_2_PO_4_/7 mM EDTA, pH 7.5), 0.4 mM NADPH and 0.5 U/L glutathione reductase suspension, were added. Determinations were performed in triplicates. The reaction was started by adding 5 µL of 24 mM DTNB in methanol. The absorption at *λ* = 412 nm was measured every 60 s for 5 min.

For determination of GSSG a control (20 μM GSSG, 160 μM GSH) was treated the same way as the samples. 125 µL sample, control, GSSG standards and blank, respectively, were combined with 5 µL 2-vinylpyridine (Sigma Aldrich, Steinheim, Germany). 20 µL of 7.4 mM triethylamine was pipetted to the upper rim of the vessel such that after mixing the pH value was between 7 and 7.5 and reaction was allowed to proceed in the thermomixer at 600 rpm for 1 h at 26 °C in order to derivatize the GSH in the sample. The determination of GSSG was then performed in analogy to the determination of tGSH described above. The absorption at *λ* = 412 nm was measured every 60 s for 10 min.

### Receptor-mediated mechanisms

#### Aryl hydrocarbon receptor (AhR), constitutive androstane receptor (CAR) and pregnan X receptor (PXR): Alkoxyresorufin O-dealkylase assays

The alkoxyresorufin dealkylase (AROD) assays were based on the method by Burke et al. (1994).

The frozen livers and lungs of the NVP-treated male Wistar rats were thawed and washed with isotonic (0.9%) NaCl solution. The tissues were ice-cooled homogenized (1 g tissue: 3 mL 0.25 M saccharose in 1 mM disodium EDTA, pH 7.4) and centrifuged (9000 g, 15 min, 4 °C). The supernatant fraction was centrifuged (100 000 *g*, 60 min, 4 °C). The resulting supernatant fraction was used for the palmitoyl-CoA-oxidase assay described further down. The pellet was taken up in 150 mM aqueous KCl solution, recentrifuged (100 000 *g*, 60 min, 4 °C) and the pellet taken up in 1 mL/g liver or 0.5 mL/g lung resuspension buffer (1 mM GSH, 1 mM disodium EDTA, 4 mM MgCl_2_, 0.1 M KH_2_PO_4_, pH 7.5 and 4% glycerol). Aliquots were stored at − 80 °C.

For determination of the enzyme activities in triplicates, 85 µL master mix (100 mM TrisHCl pH 7.5, 2 mM 5’AMP, 5 mM G-6-P, 10 U G-6-P-DH, 10 mM Dicumarol, 10 mM MgCl_2_, either 2 µM EROD or 10 µM PROD or 5 µM BROD) was added to 10 µL microsomal fraction (1 mg protein/mL). The reaction was started by the addition of 5 µL 2 mM NADPH (dissolved in 100 mM TrisHCl pH 7.5) followed by incubation at 37 °C for 16 min. The activity was determined by measuring the increase of the fluorescence (excitation at 550 nm, emission at 585 nm) due to resorufin formation compared with resorufin standards, which had been prepared by dissolving tenfold concentrated solutions in DMSO, which were then diluted tenfold in 100 mM TrisHCl pH 7.5.

As a method control the described AROD assay was performed with microsomal fractions derived from liver and lung of male Wistar rats which had been treated *i.p.* for 5 days with aroclor 1254 (500 mg/kg bw). These control assays were performed in presence of 1 μM, 10 μM or 100 μM NVP, respectively, in order to check for potential interactions of NVP with the substrate turnover in the AROD assay.

#### Peroxisome proliferator-activated receptor α (PPARα):

##### a) Lauric acid hydroxylase assay

^14^C-labelled lauric acid (55 mCi/mmol) dissolved in ethanol (0.1 mCi/mL; total volume 2.5 mL) was purchased from American Radiolabeled Chemicals. 10 µL of this solution were diluted with methanol to 2 mL. The purity was checked by radio-HPLC. For the enzyme activity determinations 1.21 mL of this solution were treated in the thermomixer at 40 °C and 300 rpm until the ethanol was completely evaporated. The residue was taken up in 550 µL DMSO and checked on the incubation days by radio-HPLC and by liquid scintillation counting (LSC). To this end the just-mentioned solution in DMSO (nominally 8.14 MBq/mL) was 100-fold diluted with methanol, 50 µL of the resultant solution used for the HPLC analysis, 100 µL for the LSC analysis (488,400 dpm/100 µL).

For the determination of the lauric acid hydroxylase activity the liver microsomal fractions (prepared as described in the preceding chapter “Aryl hydrocarbon receptor”) derived from the NVP-treated (and untreated control) male rats were adjusted with TrisHCl pH 7.5–20 mg protein/mL. Obtained samples were measured in duplicates. Controls were heat-denatured samples (15 min at 99 °C in the thermomixer at 450 rpm), zero-time samples (reaction stopped immediately after adding substrate) and buffer controls (TrisHCl pH 7.5, no microsomal protein). 155 µL master mix (100 mM TrisHCl pH 7.5, 10 mM MgCl_2_, 2 mM 5’AMP-Na_2_, 5 mM G-6-P, 10 U G-6-P-DH) and 30 µL sample were preincubated for 3 min at 37 °C. Subsequently 10 µL of a 2-mM NADPH solution and 5 µL ^14^C-lauric acid (8.14 MBq/mL) were added (lauric acid final concentration in the incubation 100 µM). The reaction was run for 20 min at 37 °C in the thermomixer (450 rpm) and stopped by the addition of 200 µL ice-cold acetone. Proteins were sedimented by centrifugation (6000 g, 10 min, 4 °C) and the supernatant used for the analysis by HPLC (system: Agilent 1260 infinity, column: EC250/4 Nucleosil, 120–5 C18, radio detector: Berthold FlowStar LB 513) under the following conditions: Injection volume 50 µL; eluent A: Methanol/acetic acid/water (600/5/400 mL), eluent B: Methanol; gradient: 0–10 min 100% eluent A, 10–20 min 0–100% eluent B, 20–30 min 100% eluent B, 30–40 min 100% eluent A; flow rate: 0.8 mL/min. In order to ascertain the assignment of the peaks to 11-hydroxylauric acid *versus* 12-hydroxylauric acid the retention times and mass spectra (HPLC-QTOF/MS) of a 12-hydroxylauric acid standard were compared with the lauric acid incubates (parameters of the HPLC-QTOF/MS system: (U)HPLC: Agilent 1200 und 1290; MS instrument: MS324/Agilent/S/N US10318001; radio detector: Raytest/Mirastar Masshunter Data Acquisition B.06.01; ionisation mode: ESI, negative mode).

##### b) Palmitoyl-CoA oxidase assay

Cyanide-insensitive palmitoyl-CoA oxidase was determined essentially according to the method by Lazarow (1981), specifically as follows:

Five µL of the liver cytosol (prepared as described in the preceding chapter “Aryl hydrocarbon receptor”) derived from the NVP-treated (and untreated control) male rats were mixed in a centrifugal analyzer Cobas Fara II (Hoffmann La Roche) with 250 µL reaction mixture consisting of 28.2 mL 50 mM Tris buffer pH 8.01, 150 µL 2% Triton-X-100, 300 µL 1 mM FAD, 300 µL 10 mM CoA, 150 µL 1.5% BSA, 90 µL 0.33 mM dithiothreitol, 300 µL 20 mM NAD and after homogenization 300 µL 100 mM KCN. The reaction was started by adding 5 µL of 5.03 µM palmitoyl-CoA aqueous solution. The increase of the absorption at *ʎ* = 334 nm due to NADH formation was determined and translated to palmitoyl-CoA oxidase U/L by the Cobas Fara II software.

### Protein quantification and determination of levels of detection (LOD) and levels of quantitation (LOQ)

Protein was determined according to the method of Bradford (1976) using bovine serum albumin as standard except for the palmitoyl-CoA-oxidase assay where the Biuret method was used according to Gornall et al. (1949).

The limit of detection (LOD) was determined by adding 3 standard deviations (SDs) to the mean of the individual results. The limit of quantification was determined by multiplying the LOD by 2 (Gottwald 2000).

### Histological investigations of the liver

#### Hematoxylin–eosin (HE) staining

HE staining was performed essentially according to Romeis (2010), specifically under the following conditions:

Parts of the frozen (− 80 °C) livers from rats treated with 0, 5, 10 or 20 ppm NVP were thawed in formalin (specifically 4% formaldehyde in sodium phosphate buffer pH 7) and left therein for fixation at least 7 days. Subsequently the livers were cut into approximately 4 mm-thick slices, which were dehydrated in increasingly concentrated ethanol (70–100%), washed in xylene and then transferred into fluid paraffin. The livers were put into kassettes with the parts which were foreseen for cutting toward the bottom, the kassettes filled with paraplast (melting point 58–62 °C) and the material left for hardening. From the embedded livers 3- to 6 µm-thick slices were cut by a rotation microtome, then stretched (in a water bath at 45 °C), transferred to a slide and dried overnight at 50 °C. Subsequently the slices were stained with HE and covered with Pertex. The slices were then microscopically evaluated. Structural changes were classified into severity grades 0, 1, 2 and 3.

#### Oil Red O (ORO) staining

ORO staining was performed essentially according to Lillie and Ashburn (1943), specifically under the following conditions:

Parts of the frozen (− 80 °C) livers from rats treated with 0, 5, 10 or 20 ppm NVP were soaked in tap water and then thoroughly frozen at − 40 °C. Subsequently they were cut at -10 to -15 °C into slices of 12–20 µm and stored in water until starting the staining procedure. The slices were then left for approximately 5 min in 60% isopropanol, subsequently stained for 10–12 min in ORO solution (5 g ORO dissolved in 1 L isopropanol, to this solution added 400 mL H_2_O, left at room temperature for 24 h and then filtered) and subsequently differentiated by immersing 2–3 times into 60% isopropanol. Afterwards they were left in water until they collected at the surface. Finally the slices were stained for 3 min in hematoxylin solution (1 g hematoxylin, 50 g KAl(SO_4_)_2_, 50 g chloral hydrate, 1 g citric acid/L H_2_O, then for maturation 0.2 g NaIO_3_/L). Subsequently they were left at least 10 min in water. Finally, the slices were transferred to microscope slides and covered with 40–60 °C warm Kaiser’s glycerol gelatin.

#### Gene expression analysis in cultivated rat hepatocytes

Isolation and cultivation of primary rat hepatocytes was performed according to a published standard operation procedure (Godoy et al. 2013) and Affymetrix gene array analysis as described in Grinberg et al. (2018). Briefly, male Wistar rats with a body weight of 220–300 g were purchased from Charles River (Sulzfeld, Germany). The animals had free access to food (sniff, Soest, Germany) and water and were kept under controlled temperature (18–26 °C), humidity (30–70%) and lighting (12 h light/dark circle). Prior to any experimental procedure, the animals were acclimated for a minimum of 6 days. This study was approved by the local committee for the welfare of experimental animals and was performed in accordance with national legislation. RNA was extracted from cultivated primary hepatocytes as described by Heise et al. (2012). Incubation of the cultivated rat hepatocytes with the test compounds was performed for 24 h. Rat hepatocytes were cultivated as sandwich cultures using 6well dishes. Three independent incubations with the test compound were performed. For Affymetrix gene chip analysis samples isolated from the 6-well dishes were stored in RNAprotect reagent from Qiagen until isolation of RNA. The RNA was quantified using a NanoDrop N-1000 spectrophotometer, and the integrity of RNA was confirmed with a standard sense automated gel electrophoresis system. Samples were used for transcriptional profiling only when their RNA quality indicator (RQI) number was > 8. First-strand cDNA was synthesized from 100 ng total RNA using an oligo-dT primer with an attached T7 promoter sequence, followed by the complementary second strand. The double-stranded cDNA molecule was used for in vitro transcription (IVT, standard Affymetrix procedure) using Genechip 3′ IVT Express Kit. During synthesis of the aRNA (amplified RNA, also commonly referred to as cRNA), a biotinylated nucleotide analogue was incorporated, which served as a label for the message. After amplification, aRNA was purified with magnetic beads and 15 µg of aRNA was fragmented with the fragmentation buffer as per the manufacturer’s instructions. Then 12.5 µg fragmented aRNA was hybridized with Affymetrix Rat Genome 230 2.0 Arrays. The chips were placed in a GeneChip Hybridization Oven-645 for 16 h at 60 rpm and 45 °C. For staining and washing, Affymetrix HWS kits were used on a Genechip Fluidics Station-450. For scanning, the Affymetrix GeneChip Scanner-3000-7G was used, and the image and quality control assessments were performed with Affymetrix GCOS software. All reagents and instruments were acquired from Affymetrix (Affymetrix, Santa Clara, CA, USA). The generated CEL files were used for further statistical analysis as described in Grinberg et al., 2018).

## Results

### Exposure

Male and female Wistar rats were exposed by inhalation to NVP nominal concentrations equal to those which had been used in the carcinogenicity bioassay by Klimisch et al. (1997a), 0, 5, 10 and 20 ppm, respectively. Table 2 shows that in the present experiments the achieved concentration levels in the inhalation chambers came satisfactorily close to the intended nominal concentrations: The mean concentrations averaged over the entire exposure period from day 1 to day 5 corresponded to the intended concentrations by ≥ 92%.

**Table 2:** Achieved NVP concentrations in the inhalation chambers

Exposure day	Intended concentrations		
	5 ppm	10 ppm	20 ppm
	Achieved concentrations		
0	4.40 ± 0.3	9.10 ± 1.1	17.90 ± 0.8
1	2.90 ± 1.3	6.40 ± 1.6	16.30 ± 2.3
2	4.30 ± 0.2	760 ± 0.7	15.60 ± 1.8
3	5.80 ± 0.1	11.70 ± 0.6	21.30 ± 0.1
4	5.80 ± 0.2	12.10 ± 0.6	20.50 ± 2.4
5	5.30 ± 1.3	11.30 ± 1.7	22.20 ± 0.7

### Clinical observations

None of the exposed rats died during the 5-day exposure. Only light clinical effects were noted: Red nose effluent (in 5 of 48 animals on days 4 or 5 of exposure), slightly increased salivation (in 1 of 48 animals on day 2 of exposure). Body weight increase was reduced in the mid-dose (10 ppm) males (*p* ≤ 0.05), and body weights were decreased in both genders (− 2.6 ± 2.5% and − 3.2 ± 2.6%, respectively) at the top dose (20 ppm) (*p* ≤ 0.01, 2-sided Dunnett test).

### Histology

**Fig. 2:**
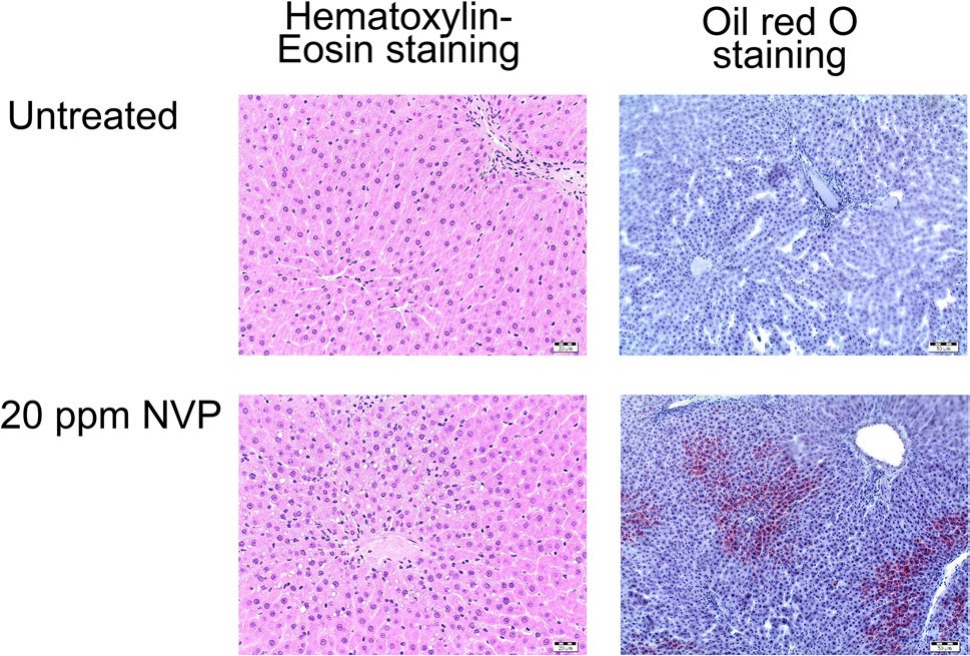
Histological examination of NVP-treated versus untreated rat liver

Histological examinations were not planned in this study, but in some livers of the males high-dose group there were already macroscopically some light spots visible. Therefore, the livers of three animals per dose and gender were histologically examined by hematoxylin–eosin staining. In most livers of the NVP-exposed rats centrilobular hepatocellular fatty change was noticed (but in none of the controls) (Fig. 2). This was the case in all males, except two of three investigated males in the low dose (5 ppm) group. In the intermediate dose (10 ppm) group, two of three males showed low-grade (grade 1) centrilobular hepatocellular fatty change, and in the high-dose (20 ppm) group, two of three males showed high-grade (grade 3) centrilobular hepatocellular fatty change. Females showed weaker effects: In one of three females of the high-dose group there was grade 1 centrilobular hepatocellular fatty change, and in one female of the high-dose group there was grade 2 centrilobular hepatocellular fatty change.

Lipid-staining (oil red O staining) of centrilobular hepatocellular fatty change was positive for only one male and one female animal in the high dose group, respectively.

### Genotoxicity

#### Micronucleus test

The micronucleus test was performed according to the OECD guideline 474 in erythrocytes obtained from the femora of rats, which had been exposed during the 5 preceding days to NVP (via inhalation) or to the positive control substance, the known genotoxin EMS (ethyl methanesulfonate, 200 mg/kg body weight perorally applied). 2000 PCEs were analyzed for micronuclei per animal.

PCE of the vehicle-treated negative control animals had 1.8 ± 1.1‰ (males) and 2.3 ± 0.6‰ (females) micronuclei, corresponding to 2.1 ± 0.9‰ regardless of gender. This is not significantly different from the historical controls of the performing laboratory (0.7–3.6‰) (obtained after inhalation or peroral treatment, which were not significantly different from each other). The number of micronuclei in the erythrocytes obtained from the positive controls, which had been treated with 200 mg/kg body wight of EMS, was 14.5 ± 6.0‰ in the erythrocytes obtained from males and 11.8 ± 2.3‰ in those obtained from females (13.2 ± 4.6‰ independent of gender) statistically significantly different from the negative controls (Wilcoxon-Test, *p* ≤ 0.01). The micronuclei observed in the EMS-treated positive control animals corresponded to the historical positive controls observed in the performing laboratory (6.1–21.9‰ after treatment with 200–300 mg EMS /kg body weight).

After treatment of the rats with 5 ppm, 10 ppm or 20 ppm NVP no significant increase in micronuclei in the PCE was observed. The mean number of micronuclei in NVP-exposed male and female rats varied from 1.4 to 2.9‰ (Table 3), which was within the range of the negative (vehicle) controls of the present study as well as of the historical negative controls.

Thus, the doses which were carcinogenic in the rats did not lead to an increase in micronuclei in the rat bone marrow. Hence, no indication on a genotoxic (clastogenic or aneugenic) potential of NVP was apparent in the micronucleus test.

In addition, in normochromatic erythrocytes (NCE) no micronuclei were observed (neither in the vehicle-treated negative controls nor after exposure to NVP). This also corresponds to the historical negative controls in which maximally one micronucleus had been observed per 10 000 NCE.

The ratio of PCE to NCE was decreased by 10% in the high-dose males (Table 3) which suggests (but is not sufficient to unambiguously prove) toxicity-dependent perturbation of the maturation of PCE to NCE and, hence, of successful transport of NVP (or toxic metabolite(s) derived from it) to the bone marrow.

**Table 3:** Micronuclei in bone marrow after treatment of rats with NVP or the positive control substance EMS

NVP dose		MN/ 1000 PCE		PCE/1000 Erythrozyten Mean ± SD	PCE/NCE quotient (%)
		Mean ± SD	Range		
0 ppm	M	1.8 ± 1.1	0.0–3.0	609 ± 29	100
	F	2.3 ± 0.6	1.5–3.0	636 ± 48	100
5 ppm	M	2.6 ± 0.9	1.5–4.0	609 ± 40	100
	F	1.4 ± 0.7	0.5–2.5	629 ± 18	99
10 ppm	M	2.9 ± 1.9	1.5–6.5	622 ± 33	102
	F	1.7 ± 1.0	0.5–3.0	612 ± 44	96
20 ppm	M	2.0 ± 0.9	0.5–3.0	553 ± 57	90
	F	2.3 ± 0.8	1.0–3.0	615 ± 48	97
EMS	M	14.5 ± 6.0	3.0–20.0	616 ± 50	101
	F	11.8 ± 12.3	9.0–14.5	563 ± 29	89

*NVP*, *N*-vinyl pyrrolidone; *MN*, micronuclei; *PCE*, polychromatic erythrocytes; *NCE*, normochromatic erythrocytes; *SD*, standard deviation; *EMS*, ethyl methanesulfonate (200 mg/kg body weight)

#### Comet assay

After exposure of the rats to NVP the Comet assay was performed in the lung, the organ of first contact with NVP, and in the liver, the target organ of the NVP-induced carcinogenicity of NVP.

Since cytotoxicity could interfere with the Comet assay and with the results obtained by its use and since the cells may be damaged by the procedures, their viabilities were determined by the Trypan blue assay. No cytotoxicity was apparent: the viabilities of the negative vehicle controls were 94 ± 2% und 95 ± 3% for the liver cells and 98 ± 1% und 99 ± 1% for the lung cells of male and female rats, respectively (range of the individual animals 91 to 100% for liver cells and between 97 and 100% for lung cells). Exposure to NVP did not decrease these viabilities, for individual animals lying between 90 and 99% for liver cells and between 93 and 100% for lung cells (range of the individual animals 90 to 99% for liver and 93 to 100% for lung cells (Tables 4, 5, 6, 7). Thus, viabilities were satisfactory for the target organ liver and for the organ of first contact, the lung, and no influence of the gender nor of the exposure to NVP nor of the technical procedure of cell isolation was apparent. All of this was also true for the exposure to the positive control compound, EMS (Tables 4, 5, 6, 7).

**Table 4:** Comet assays^a^ in the liver of male Wistar rats

NVP dose	Ani-mal (number)	Indi-vidual viability (%)	Viability (%) (mean ± SD)	*Hedgehogs*/100 cells (mean ± SD)	TI (%)^b^	TI (%) (mean ± SD)
0 ppm	1	91	94 ± 2	3 ± 2	0.3	0.7 ± 0.34
	2	96			1.1	
	3	96			0.6	
	4	94			1.1	
	5	96			0.7	
	6	93			0.5	
5 ppm	7	90	95 ± 3	3 ± 2	1.1	0.9 ± 0.22
	8	92			1.2	
	9	96			0.9	
	10	96			0.6	
	11	98			0.7	
	12	96			0.9	
10 ppm	13	96	96 ± 1	3 ± 1	0.7	1.0 ± 0.68
	14	97			2.4	
	15	96			0.7	
	16	98			1.0	
	17	96			0.6	
	18	94			0.7	
20 ppm	19	98	97 ± 1	3 ± 1	1.2	0.7 ± 0.34
	20	95			0.8	
	21	99			0.5	
	22	95			0.5	
	23	98			1.0	
	24	96			0.3	
EMS^c^ (Positivecontrol)	49	91	95 ± 3	8 ± 2	18.9	18.6 ± 5.97
	50	94			17.5	
	51	95			29.0	
	52	96			10.6	
	53	99			19.3	
	54	94			16.4	

^a^Conventional assays (no FPG added)

^b^Mean of medians from 2 slides, each carrying 50 evaluated cells

^c^*EMS*, ethyl methanesulfonate

**Table 5:** Comet assays^a^ in the liver of female Wistar rats

NVP dose	Ani-mal (number)	Indi-vidual viability (%)	Viability (%) (mean ± SD)	*Hedgehogs*/100 cells (mean ± SD)	TI (%)^b^	TI (%) (mean ± SD)
0 ppm	25	91	95 ± 3	4 ± 2	1.0	0.6 ± 0.30
	26	100			0.7	
	27	98			0.4	
	28	96			0.9	
	29	93			0.6	
	30	94			0.2	
5 ppm	31	98	97 ± 1	3 ± 3	0.6	1.0 ± 0.40
	32	98			0.9	
	33	96			1.1	
	34	99			1.7	
	35	97			0.8	
	36	95			0.8	
10 ppm	37	96	96 ± 1	4 ± 4	1.3	0.9 ± 0.48
	38	99			0.6	
	39	95			0.5	
	40	97			1.4	
	41	94			1.3	
	42	96			0.3	
20 ppm	43	96	97 ± 1	3 ± 2	0.7	0.7 ± 0.34
	44	97			1.3	
	45	97			0,5	
	46	98			0,5	
	47	96			1,0	
	48	95			0.4	
EMS^c^ (Positivecontrol)	55	99	96 ± 2	7 ± 4	9.1	16.3 ± 4.49
	56	95			13.6	
	57	96			17.5	
	58	95			22.4	
	59	97			17.8	
	60	97			17.3	

^a^conventional assays (no FPG added)

^b^Mean of medians from 2 slides, each carrying 50 evaluated cells

^c^*EMS*, ethyl methanesulfonate

**Table 6:** Comet assays^a^ in the lung of male Wistar rats

NVP dose	Ani-mal (number)	Indi-vidual viability (%)	Viability (%) (mean ± SD)	*Hedgehogs*/100 cells (mean ± SD)	TI (%)^b^	TI (%) (mean ± SD)
0 ppm	1	97	98 ± 1	6 ± 3	1.2	1.2 ± 0.47
	2	99			1.0	
	3	99			2.1	
	4	99			0.8	
	5	98			1.0	
	6	98			0.9	
5 ppm	7	98	98 ± 1	4 ± 3	1.9	1,1 ± 0.61
	8	97			1.8	
	9	99			0.6	
	10	99			0.7	
	11	97			0.7	
	12	100			0.8	
10 ppm	13	99	98 ± 2	7 ± 5	5.7	1.8 ± 1.94
	14	99			1.3	
	15	98			1.2	
	16	99			0.7	
	17	93			1.0	
	18	99			1.1	
20 ppm	19	100	99 ± 1	3 ± 1	0.4	0.6 ± 0.28
	20	100			0.9	
	21	98			0.9	
	22	97			0.6	
	23	99			0.3	
	24	100			0.4	
EMS^c^ (Positivecontrol)	49	99	99 ± 1	9 ± 4	20.7	19.3 ± 5.15
	50	99			14.6	
	51	99			26.5	
	52	100			13.1	
	53	99			23.3	
	54	98			17.8	

^a^conventional assays (no FPG added)

^b^mean of medians from 2 slides, each carrying 50 evaluated cells

^c^*EMS*, ethyl methanesulfonate

Hedgehogs (comets with no or small head and long, diffuse tail), indications of cytotoxicity or extreme genotoxicity, were, compared with the negative (vehicle) controls not significantly increased in the lung or liver cells of both genders after treatment of the animals with NVP. Hedgehogs amounted in the NVP-treated lung cells to 1–7% and in the NVP-treated liver cells to 1–5% compared with vehicle-treated controls (Tables 4, 5, 6, 7). This was independent of performing the Comet assay in presence or absence of the DNA repair enzyme *N*-formyl pyrimidine glycosylase (NPG). However, treatment of the animals with the positive control substance, EMS, led in the assays in the conventional Comet assays (no FPG added) to approximately twofold increases of the number of hedgehogs: averaged for both genders from 3 5 to 7.5 and from 4.5 to 9 per 100 cells in the liver and lung cells, respectively (Tables 4, 5, 6, 7). In presence of FPG only hedgehogs were seen after treatment with EMS.

As measure for DNA damage the tail intensity (TI) (percent DNA in the tail) was chosen, since on grounds of its independence of the instruments and software used it is generally preferred and recommended by the OECD guideline 489.

**Fig. 3:**
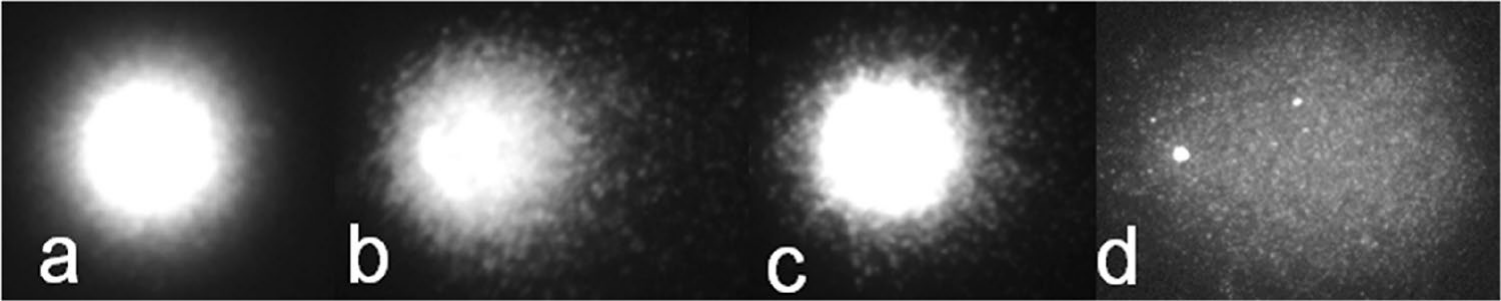
Rat liver cells in Comet assay. **a**, **c** vehicle control, **b**, **d** after treatment of the animals with the positive control substance, ethyl methanesulfonate (EMS). **a**, **b** Comet assay in absence of formyl pyrimidine glycosylase (FPG). **c**, **d** Comet assay in presence of FPG

The positive control substance (200 mg EMS/kg body weight) led in the conventional Comet assay (no FPG added) compared with the negative vehicle-control to statistically significant (*p* ≤ 0.01) and marked increases of the TI. These amounted in the liver cells to 27 and 28-fold in male and female animals, respectively and in the lung to 17 and 21-fold in male and female animals, respectively (Tables 4, 5, 6, 7). In the presence of FPG it was not possible to numerically define the increase of the TI after the treatment with EMS, but image analysis convincingly showed a marked increase (Fig. 3c, d).

In contrast to these marked increases upon treatment of the animals with the positive control substance EMS the treatment with NVP did not lead to significant increases as observed using the conventional Comet assay (no FPG added) (Tables 4, 5, 6, 7) nor in presence of FPG (Tables 8, 9, 10, 11). This was true for both genders in the liver (Tables 4, 5, 8, 9) and in the lung (Table 6, 7, 10, 11).

**Table 7:** Comet assays^a^ in the lung of female Wistar rats

NVP dose	Ani-mal (number)	Indi-vidual viability (%)	Viability (%) (mean ± SD)	*Hedgehogs*/100 cells (mean ± SD)	TI (%)^b^	TI (%) (mean ± SD)
0 ppm	25	99	99 ± 1	3 ± 2	0.5	0.9 ± 0.30
	26	100			1.1	
	27	100			1.2	
	28	100			0.6	
	29	100			1.3	
	30	97			1.0	
5 ppm	31	99	99 ± 1	2 ± 1	1.3	1.2 ± 0.34
	32	99			0.7	
	33	98			1.2	
	34	99			1.5	
	35	98			1.6	
	36	98			1.2	
10 ppm	37	98	98 ± 1	2 ± 2	0.4	0,. ± 0.24
	38	97			0.9	
	39	99			0.9	
	40	97			0.9	
	41	99			0.5	
	42	100			0.6	
20 ppm	43	98	98 ± 1	2 ± 3	0.7	1.0 ± 0.32
	44	98			0.9	
	45	99			0.7	
	46	96			1.2	
	47	98			1.0	
	48	98			1.6	
EMS^c^ (Positivecontrol)	55	99	99 ± 1	9 ± 5	13.2	16.7 ± 2.83
	56	99			13.0	
	57	99			18.7	
	58	97			18.8	
	59	100			18.7	
	60	98			17.8	

^a^conventional assays (no FPG added)

^b^mean of medians from 2 slides, each carrying 50 evaluated cells

^c^*EMS*, ethyl methanesulfonate

**Table 8:** Comet assays in presence of FPG^a^ in the liver of male Wistar rats

NVP dose	Ani-mal (number)	Indi-vidual viability (%)	Viability (%) (mean ± SD)	*Hedgehogs*/100 cells (mean ± SD)	TI (%)^b^	TI (%) (mean ± SD)
0 ppm	1	97	98 ± 1	1 ± 1	9.8	6.2 ± 2.29
	2	99			6.0	
	3	99			5.4	
	4	99			5.4	
	5	98			7.7	
	6	98			3.1	
5 ppm	7	98	98 ± 1	1 ± 1	5.7	5.3 ± 1.09
	8	97			4.3	
	9	99			5.8	
	10	99			5.2	
	11	97			4.0	
	12	100			7.0	
10 ppm	13	99	98 ± 2	2 ± 1	8.3	6.1 ± 1.67
	14	99			7.5	
	15	98			3.7	
	16	99			4.9	
	17	93			6.5	
	18	99			5.8	
20 ppm	19	100	99 ± 1	2 ± 1	6.8	5.0 ± 1.09
	20	100			4.4	
	21	98			5.0	
	22	97			4.7	
	23	99			3.6	
	24	100			5.6	

^a^FPG, formamido pyrimidine DNA glycosylase

^b^mean of medians from 2 slides, each carrying 50 evaluated cells

**Table 9:** Comet assays in presence of FPG^a^ in the liver of female Wistar rats

NVP dose	Ani-mal (number)	Indi-vidual viability (%)	Viability (%) (mean ± SD)	*Hedgehogs*/100 cells (mean ± SD)	TI (%)^b^	TI (%) (mean ± SD)
0 ppm	25	99	99 ± 1	5 ± 3	7.4	7.5 ± 1.13
	26	100			7.8	
	27	100			7.2	
	28	100			8.3	
	29	100			8.7	
	30	97			5.5	
5 ppm	31	99	99 ± 1	4 ± 2	7.1	7.6 ± 1 + .47
	32	99			6.5	
	33	99			8.4	
	34	99			9.0	
	35	98			9.3	
	36	98			5.6	
10 ppm	37	98	98 ± 1	3 ± 2	5.5	7.9 ± 2.74
	38	97			10.7	
	39	99			5.6	
	40	97			5.3	
	41	99			9.4	
	42	100			11.0	
20 ppm	43	98	98 ± 1	4 ± 2	9.2	7.0 ± 1.97
	44	98			5.2	
	45	99			8.3	
	46	95			6.3	
	47	98			8.6	
	48	98			4.4	

^a^FPG, formamido pyrimidine DNA glycosylase

^b^mean of medians from 2 slides, each carrying 50 evaluated cells

**Table 10:** Comet assays in presence of FPG^a^ in the lung of male Wistar rats

NVP dose	Ani-mal (number)	Indi-vidual viability (%)	Viability (%) (mean ± SD)	*Hedgehogs*/100 cells (mean ± SD)	TI (%)^b^	TI (%) (mean ± SD)
0 ppm	1	97	98 ± 1	3 ± 2	9.6	9.4 ± 2.04
	2	99			12.1	
	3	99			8.5	
	4	99			8.5	
	5	98			11.2	
	6	98			6.4	
5 ppm	7	98	98 ± 1	2 ± 2	6.5	6.4 ± 0.69
	8	97			6.1	
	9	99			7.5	
	10	99			6.1	
	11	97			6.6	
	12	100			5.5	
10 ppm	13	99	98 ± 2	4 ± 4	12.0	7.9 ± 3.21
	14	99			3.6	
	15	98			6.0	
	16	99			7.3	
	17	93			7.2	
	18	99			11.4	
20 ppm	19	100	99 ± 1	1 ± 1	3.8	3.9 ± 0.72
	20	100			3.5	
	21	98			5.2	
	22	97			3.6	
	23	99			3.3	
	24	100			4.2	

^a^FPG, formamido pyrimidine DNA glycosylase

^b^mean of medians from 2 slides, each carrying 50 evaluated cells

An unexpected observation was the *decrease* of the TI observed in the conventional Comet assay (no FPG added) in the male lung cells upon the 20 ppm NVP exposure and in the Comet assay variation with FPG in the male lung cells upon the 5 ppm and 20 ppm NVP exposure. Since a decrease was not observed under any other condition (not in male lung at other doses, not in male liver at any dose and not in any of the female groups, Tables 4, 5, 6, 7, 8, 9, 10, 11), the result is considered biologically irrelevant (for further discussion see “Discussion” section).

**Table 11:** Comet assays in presence of FPG^a^ in the lung of female Wistar rats

NVP dose	Ani-mal (number)	Indi-vidual viability (%)	Viability (%) (mean ± SD)	*Hedgehogs*/100 cells (mean ± SD)	TI (%)^b^	TI (%) (mean ± SD)
0 ppm	25	99	99 ± 1	3 ± 2	8.8	8.7 ± 1 + .81
	26	100			10.7	
	27	100			7.9	
	28	100			8.0	
	29	100			10.6	
	30	97			5.9	
5 ppm	31	99	99 ± 1	3 ± 2	7.3	7.8 ± 1.30
	32	99			8.6	
	33	99			7.0	
	34	99			6.8	
	35	98			10.1	
	36	98			7.1	
10 ppm	37	98	98 ± 1	1 ± 1	8.7	7.2 ± 1.81
	38	97			8.1	
	39	99			7.9	
	40	97			8.4	
	41	99			6.0	
	42	100			4.0	
20 ppm	43	98	98 ± 1	3 ± 1	6.2	7.4 ± 2.90
	44	98			8.6	
	45	99			10.2	
	46	95			2.3	
	47	98			7.7	
	48	98			9.7	

^a^FPG, formamido pyrimidine DNA glycosylase

^b^Mean of medians from 2 slides, each carrying 50 evaluated cells

Thus, no indication for an increase of the TI in the Comet assay and hence no indication of DNA strand breaks (conventional Comet assay without addition of FPG) or oxidative DNA damage or generation of apurinic sites (Comet assay in presence of FPG) upon treatment with NVP was obtained in the Comet assay at doses of NVP corresponding to those which had led to carcinogenicity (Klimisch et al. 1997a). Hence, no genotoxicity of NVP was observed in the Comet assay.

#### Oxidative stress

Since genotoxicity does not appear to be causative for the carcinogenicity of NVP (see above), oxidative stress, favoring oxidative damage to biological macromolecules including DNA, proteins and lipids, was investigated as a possible alternative mechanism leading to carcinogenicity.

To this end the concentration of (reduced) glutathione (GSH), the major biological antioxidant of the target tissue for the NVP-induced carcinogenicity, the liver, as well as its oxidized form, GSSG, and the sum of the two, “total glutathione” (tGSH), were determined in the liver of the NVP-exposed rat, the species in which the NVP hepatocarcinogenicity had been discovered (Klimisch et al. 1997a). The non-protein-bound sulfhydryl residues (NPSH), which in the rat liver consist mostly of GSH, were determined as approximation for the GSH concentration, while GSSG and tGSH concentrations were measured by the GSH recycling assay.

Table 12 shows the LOD, LOQ and recovery of the procedures used. Taking the dilution (20-fold) and the weight of the liver equivalent used (0.111 g/mL) into account it follows from the data in Table 12 (LOQ 144 nM) that 26 nmol GSSG/g liver correspond to the LOQ. Table 12 shows an LOQ of 98 nM tGSH in the well, which taking into account the dilution (assay 40-fold, sample fourfold) in this experiment and the weight of the liver equivalent used allows a quantification of GSH down to 140 nmol GSH/g liver. In the NPSH-assay the LOQ was 2.1 µM in the well corresponding to 230 nmol/g liver, hence slightly higher than in the recycling assay, but in the same order of magnitude.

The precision of the procedures used were examined by determination of the recoveries. The mean recovery of GSH was 104% in the recycling assay and approximately 98% in the NPSH assay. In order to ensure that GSH and GSSG present in the recycling assay do not influence their determination a control solution was analyzed which contained at a tGSH concentration of 200 µM 10% of GSSG, since this represents approximately the ratio expected in the liver samples. In these control solutions the calculated mean recoveries were 99% for tGSH and 106% for GSSG (Table 12).

Exposure of the rats to NVP led to concentrations of GSH 3.72–5.23 µmol/g liver in the NPSH assay and to 3.06–5.48 µmol/g liver in the GSH recycling assay compared with control values of 3.76–5.10 µmol/g liver and 3.32–5.01 µmol/g liver in these two assays, respectively, upon exposure to vehicle only (Table 13). Mean GSSG concentrations were after treatment of the animals with 5 ppm NVP at 116 ± 32 nmol/g liver, after treatment with 10 ppm at 135 ± 12 nmol/g liver, after treatment with 20 ppm at 136 ± 22 nmol/g liver compared with 108–163 nmol/g liver in the vehicle control (Table 13).

**Table 12:** LOD, LOQ and recovery of the GSH and GSSG test procedures used

Test system		LOD (nM)	LOQ (nM)	*c*_nominal_ (µM)	C_determinedt_ (µM)	Recovery (%)
Glutathione recycling assay	tGSH	49 (*n* = 12)	98	640	659–680 (*n* = 3)	103–106 (*n* = 3)
				200	181–216 (*n* = 6)	90–108 (*n* = 6)
	GSSG	72 (*n* = 12)	144	20	18,9–24,1 (*n* = 8)	94–120 (*n* = 8)
NPSH	GSH	1041 (*n* = 27)	2082	480	470,0 ± 28,3 (*n* = 3)	91–102 (*n* = 3)

*LOD*, limit of detection; *LOQ*, limit of quantification; *GSH*, glutathione (reduced); *GSSG*, oxidized glutathione; *tGSH*, total glutathione (sum of GSH + GSSG); *NPSH*, non-protein sulfhydryl; *C*, concentration

**Table 13:** Glutathione levels after treatment of the rats with NVP^a^

NVP dose	Animal (number)	tGSH^b^ (µmol/g liver)		GSSG^b^ (nmol/g liver)			GSH^b,c^ (µmol/g liver)			NPSH^b^ (µmol/g liver)	
		Individual animal	Group	Individual animal		Group	Individual animal		Group	Individual animal	Group
0 ppm	1	4.80	4.69 ± 0.71	145		137 ± 21	4.51		4.42 ± 0.70	4,82	4.52 ± 0.55
	2	5.32		154			5.01			5,02	
	3	4.47		163			4.15			4,11	
	4	3.54		108			3.32			3,76	
	5	5.54		125			5.29			5,10	
	6	4.50		127			4.25			4,30	
5 ppm	7	4.10	4.72 ± 0.67	133		116 ± 32	3.84		4.49 ± 0.68	3,86	4.42 ± 0.52
	8	5.63		71			5.48			5,13	
	9	4.92		144			4.63			4,59	
	10	4.16		114			3.93			4,10	
	11	5.33		149			5.04			4,86	
	12	4.17		87			4.00			3,99	
10 ppm	13	3.70	4.46 ± 0.62	132		135 ± 12	3.44		4.19 ± 0.61	3,39	4.23 ± 0.58
	14	4.21		132			3.95			4.11	
	15	4.94		120			4.70			4.72	
	16	5.28		151			4.98			4.83	
	17	4.70		147			4.41			4.57	
	18	3.91		126			3.65			3.76	
20 ppm	19	4.75	4.20 ± 0.65	151		136 ± 22	4.45		4.04 ± 0.62	4.47	4.44 ± 0.55
	10	4.83		130			4.57			5.23	
	21	3.27		106			3.06			3.72	
	22	3.77		142			3.48			3.88	
	23	4.55		121			4.31			4.67	
	24	4.68		167			4.35			4.64	

^a^Mean ± standard deviation

^b^*tGSH*, total glutathione; *GSSG*, oxidized glutathione; *GSH*, reduced glutathione; *NPSH*, non-protein sulfhydryl

^c^GSH recycling assay

The proportion of reduced glutathione GSH within the total glutathione tGSH as well as the GSH/GSSG ratio was determined using the GSH recycling assay. The former amounted to 94.1% ± 0.9%, 95.0% ± 1.4%, 93.9% ± 0.8% and 93.6% ± 0.9%, the latter to 32.6 ± 5.5, 42.2 ± 18.4, 31.2 ± 4.6 and 30.0 ± 4.6 in the vehicle control and in the 5 ppm, 10 ppm and 20 ppm NVP exposure groups, respectively.

Thus, no influence of the treatment of the animals with doses of NVP corresponding to carcinogenic doses (Klimisch et al. 1997a) on the concentrations of reduced or oxidized glutathione nor on the percent contribution of reduced glutathione to the total glutathione nor on the GSH:GSSG ratio in the rat liver was apparent. Hence, no indication of a contribution of oxidative stress to the NVP induced carcinogenicity was obtained.

### Receptor-mediated mechanisms

#### Aryl hydrocarbon receptor (AhR), constitutive androstane receptor (CAR) and pregnane X receptor (PXR) (AROD assay)

In order to investigate the potential involvement of hepatic receptor activation in the hepatocarcinogenesis of NVP the activation of the aryl hydrocarbon receptor (AhR), the constitutive androstane receptor (CAR) and the pregnane X receptor (PXR) were investigated by means of a potential activity increase of the respective alkyloxy resorufin dealkylase (AROD), ethoxy resorufin deethylase (EROD), pentoxy resorufin depentylase (PROD) and benzyloxy resorufin debenzylase (BROD) of male rat liver (target organ of the NVP-mediated carcinogenesis) and lung (first contact tissue of the NVP exposure) microsomes. EROD, PROD and BROD are mediated preferentially by CYP1A, CYP2B and CYP 2B/3A and were determined using their preferential substrates 7-ethoxyresorufin, 7-pentoxyresorufin and 7-benzyloxyresorufin, respectively.

Table 14 shows the LODs and LOQs of the AROD assays in the tissues investigated and Table 15 shows that the vehicle control values for AROD activities in the present study are reasonably close to historical control values and that induction of AROD activities are still well seen after storage at -80 °C of rat liver microsomes up to 18 months.

**Table 14:** LOD and LOQ of the AROD assays in the tissues used

Assay	Organ	LOD (pmol/min/mg protein)	LOQ (pmol/min/mg Pprotein)
EROD	Liver	1,0	2,0
	Lung	1,6	3,1
PROD	Liver	3,1	6,2
	Lung	9,6	19,1
BROD	Liver	0,5	1,1
	Lung	1,8	3,6

*LOD*, limit of detection; *LOQ*, limit of quantification; *AROD*, alkyloxyresorufin dealkylase; *EROD*, 7-ethoxyresorufin deethylase; *PROD*, 7-pentoxyresorufin depentylase; *PROD*, 7-pentoxyresorufin depentylase

**Fig. 4:**
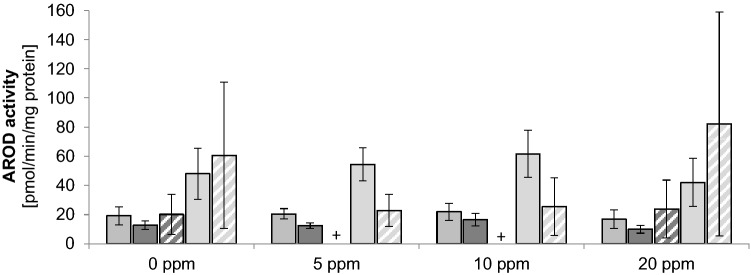
AROD activities after treatment of the rats with various doses of NVP (mean ± SD; *n* = 6). Activities are shown in the sequence EROD liver, PROD liver, PROD lung, BROD liver, BROD lung, + below LOD. *AROD*, alkyloxyresorufin dealkylase; *EROD*, 7-ethoxyresorufin deethylase; *PROD*, 7-pentoxyresorufin depentylase; *BROD*, 7-benzyloxyresorufin debenzylase

Liver microsomal EROD activities were after the treatment of the rats with 5 ppm, 10 ppm and 20 ppm NVP 20.5 ± 3.6 pmol/min/mg protein, 22.0 ± 5.8 pmol/min/mg protein and 16.9 ± 6.4 pmol/min/mg protein, respectively, compared with 19.3 ± 6.2 pmol/min/mg protein in the vehicle-treated controls (Fig. 4), hence no indication of any significant change of CYP1A activities due to the treatment with NVP. The EROD activities were about tenfold above the LOQ of 2.0 pmol/mg protein and were close to the historical controls of 22–41 pmol/min/mg protein (*n* = 7).

EROD activities in the lung microsomes were after NVP treatment and in the vehicle controls below the LOD of 1.6 pmol/min/mg protein.

Liver microsomal PROD activities were after the treatment of the rats with 5 ppm, 10 ppm and 20 ppm NVP 12.4 ± 1.9 pmol/min/mg protein, 16.5 ± 4.3 pmol/min/mg protein and 10.0 ± 2.8 pmol/min/mg protein, respectively, compared with 12.8 ± 3.0 pmol/min/mg protein in the vehicle-treated controls (Fig. 4), hence no indication of any significant change of CYP2B activities due to the treatment with NVP. All activities were above the LOQ of 6.2 pmol/min/mg protein and were in the same order of magnitude as the historical controls of 18–29 pmol/min/mg protein, albeit slightly lower.

PROD activities in the lung microsomes were below the LOD of 9.6 pmol/min/mg protein in 14 of 24 rats. It was not possible to calculate a mean PROD activity in the 5 ppm and 10 ppm NVP groups. The activities varied strongly and independently of the NVP exposure levels. The PROD activities in the high-dose (20 ppm NVP) group were not significantly different from those in the vehicle control group (Fig. 4). Hence, no indication of an influence of the NVP exposure on lung PROD activities was obtained.

Liver microsomal BROD activities were after the treatment of the rats with 5 ppm, 10 ppm and 20 ppm NVP, 54.5 ± 11.3 pmol/min/mg protein and 61.7 ± 16 pmol/min/mg protein and 42.1 ± 16.5 pmol/min/mg protein, respectively, compared with 48.1 ± 17.5 pmol/min/mg protein in the vehicle-treated controls (Fig. 4), hence no indication of any significant change of CYP3A activities due to the treatment with NVP. All activities were approximately 40–55 above the LOQ of 1.1 pmol/min/mg protein and were in the same order of magnitude as the historical controls of 63–141 pmol/min/mg protein, albeit slightly lower.

BROD activities in the lung microsomes were after the treatment of the rats with 5 ppm, 10 ppm and 20 ppm NVP 22.9 ± 11.0 pmol/min/mg protein, 25.5 ± 19.8 pmol/min/mg protein and 82.1 ± 76.8 pmol/min/mg protein, respectively, compared with 60.7 ± 50.3 pmol/min/mg protein in the vehicle-treated controls (Fig. 4), hence no indication of any significant change of CYP3A activities due to the treatment with NVP, albeit the interindividual variation was very large. All activities were above the LOQ of 3.6 pmol/min/mg protein.

It is not a priori completely excluded that some of the inhaled NVP may have bound to CYPs leading to suicide inhibition potentially masking enzyme induction. Table 16 shows that 1, 10 or 100 µM NVP does not lead to inhibition of EROD, PROD or BROD activities in Aroclor 1254-induced rat liver microsomes. All activities were above the LOQ.

**Table 15:** AROD activities of the present vehicle controls and historical controls of rat liver microsomes

Group	Substrate	Activity (pmol/min/mg protein)
Vehicle control of the present NVP inhalation study (*n* = 6)	EROD	19 ± 6
	PROD	13 ± 4
	BROD	48 ± 18
Historical controls (*n* = 7)	EROD	22–41
	PROD	18–29
	BROD	63–141
Aroclor 1254-induced control (storage < 1 month)	EROD	1070 ± 72
	PROD	195 ± 6
	BROD	892 ± 36
Aroclor 1254- induced control (storage approximately 18 months)	EROD	1155 ± 91
	PROD	126 ± 8
	BROD	772 ± 51

*AROD*, alkyloxyresorufin dealkylase; *EROD*, 7-ethoxyresorufin deethylase; *PROD*, 7-pentoxyresorufin depentylase; *PROD*, 7-pentoxyresorufin depentylase

**Table 16:** Lack of EROD, PROD or BROD inhibition by NVP in Aroclor 1254-induced rat liver microsomes

Substrate	Enzyme activity (pmol/min/mg protein)				
	LOQ (*n* = 9)	NVP concentration (µM)			
		0	1	10	100
EROD	1.70	821.5 ± 54.9	859.9 ± 14.1	827.9 ± 13.6	858.9 ± 33.9
PROD	4.84	78.9 ± 3.2	88.6 ± 3.3	92.1 ± 6.0	94.6 ± 2.3
BROD	6.46	445.1 ± 9.1	470.1 ± 29.7	445.1 ± 20.9	456.5 ± 30.9

*AROD*, alkyloxyresorufin dealkylase; *EROD*, 7-ethoxyresorufin deethylase; *PROD*, 7-pentoxyresorufin depentylase; *PROD*, 7-pentoxyresorufin depentylase; *NVP*, *N*-vinyl pyrrolidone; *LOQ*, limit of quantification

In summary, in the liver microsomes of the male rats investigated all AROD activities were clearly above the LOQ and could reliably be determined. All activities were in the same order of magnitude as the historical controls. Treatment of the rats with doses of NVP which corresponded to those which were hepatocarcinogenic (Klimisch et al 1997a) did not lead to significant changes of AROD activities preferentially mediated by CYP1A, 2B and 3A, respectively, in the cancer target organ, the rat liver.

In the microsomes of the organ of first contact of inhaled NVP, the lung, also no significant changes of AROD activities upon NVP exposure were noticed, albeit many measurements suffered from very large interindividual variations and/or very low activities, some below the LOD.

Thus, no indications of AhR, CAR or PXR activation as possibly mediating or contributing to the hepatocarcinogenicity of NVP were obtained.

### Peroxisome-proliferator-activated receptor α (PPARα)

Activation of PPARα leads to non-genotoxic hepatocarcinogenesis in rodents (Gonzales and Shaw 2008). It was, therefore, investigated whether exposure of rats to hepatocarcinogenic doses of NVP (Klimisch et al. 1997a) leads to activation of PPARα by determining CYP4A activity which is regulated by PPARα. As model reaction for CYP4A activity the 12-hydroxylation of lauric acid in the microsomal fraction from high-dose (20 ppm) NVP-exposed male rats was used. In the same assay the 11-hydroxylation of lauric acid, which is mediated by CYP2E1, can also be determined. CYP2E1 mediates the metabolic activation of many pro-carcinogens (Oesch-Bartlomowicz and Oesch 2007). It was, therefore, also quantified in the present study.

Introduction of a gradie4nt into the standard HPLC method allowed the separation of the 11-hydroxy lauric acid peak from the 12-hydroxy-lauric acid peak. Determination of LOD and/or LOQ was not necessary since all incubations led to easily quantifiable, clear peaks of hydroxylated products. In the negative controls (no protein or protein heat-denatured before or immediately after addition of substrate to the incubation mixture) no hydroxylated products were visible excluding a significant contribution of possible matrix effects or abiotic oxidation to the formation of hydroxylated lauric acid metabolites.

^14^C-lauric acid was used as substrate. Radio-HPLC confirmed its radiochemical purity and storage stability. Examination of its radioactivity by liquid scintillation counting showed a recovery of 100.4%.

Three peaks with the retention times of approximately 16.6 min, 17.4 min and 22.6 min resulted from all active incubations. ^14^C-lauric acid as reference identified the peak at 22.6 min as lauric acid. The two hydroxylated products were not available in radioactive form. Unlabeled 12-hydroxy lauric acid had a retention time of 16.1 min allowing the identification of the corresponding peak after active incubation of lauric acid. The third peak was tentatively identified as the second known hydroxylated lauric acid metabolite, 11-hydroxy lauric acid by its fragmentation pattern, which was similar to that of the 12-hydroxy lauric acid.

The enzymatic activity was expressed as the peak area divided by the incubation time (20 min) and amount of protein (in mg). This activity was 14.727 ± 5456 peak area/min/mg protein after the high-dose (20 ppm) NVP exposure of the rats compared with 10.983 ± 2236 peak area/min/mg protein vehicle-treated controls (Table 17). Hence the exposure may appear to lead to a slight increase of 1.3-fold. However, this slight increase was not statistically significant. Thus, these results do not show a NVP-induced PPARα activation. Yet, in order to go safe, these results were verified using palmitoyl-CoA-oxidase as a second marker of a PPARα-dependent enzyme activity as shown below.

**Table 17:** Lauric acid hydroxylation^a^

NVP dose	Animal (num-ber)	ω-hydroxylase (CYP4A-activity)			ω-1-hydroxylase (CYP2E1-activity)		
		Peak surface	Enzyme activity individual	Enzyme activity group	Peak surface	Enzyme activity individual	Enzyme activity group
0 ppm	1	16,449	10,966	10,983 ± 2236	6737	4491	4512 ± 1050
	2	10,791	7194		4325	2883	
	3	17,564	11,709		6828	4552	
	4	15,737	10,491		6957	4638	
	5	21,137	14,091		9280	6187	
	6	17,170	11,447		6482	4321	
20 ppm	19	27,314	20,495	14,727 ± 5456	9940	7458	5149 ± 2010
	20	28,341	20,593		10,040	7295	
	21	15,814	10,543		5283	3522	
	22	21,818	14,545		7513	5008	
	23	23,109	15,406		7824	5216	
	24	11,088	6779		3996	2393	

Mean ± standard deviation; enzyme activity represented as peak surface/min/mg protein resulting from 0.075 mg submitted to HPLC analysis

The 11-hydroxylation of lauric acid, catalyzed preferentially by CYP2E1, was putatively represented by the peak at 17.4 min retention time, which amounted to 5149 ± 2010 peak area/min/mg protein after the high-dose (20 ppm) treatment of the rats compared with 4512 ± 1050 peak area/min/mg protein in the vehicle-treated controls (Table 17) corresponding to a statistically not significant increase of 1.1-fold. Thus, no indication of a NVP-dependent increase of CYP2E1 activity was obtained.

A further marker of PPARα activation is the activity of cyanide-insensitive palmitoyl CoA oxidase/acyl CoA oxidase, the first enzyme of peroxisomal ß-oxidation.

After treatment of male rats with 5 ppm, 10 ppm or 20 ppm NVP the activities of cyanide-insensitive palmitoyl CoA oxidase in the liver cytosol were 5.43 ± 0.71, 5.47 ± 0.71 and 4.68 ± 0.71 nmol/min/mg protein, respectively, compared with 6.02 ± 0.49 nmol/min/mg protein in the vehicle-treated controls. Hence, all activities appeared reduced after NVP exposure, at the highest dose (20 ppm) even statistically significantly so. Thus, clearly there was no NVP-dependent increase. No indication for PPARα activation by NVP was obtained.

In summary, neither the results obtained with lauric acid as substrate of CYP4A nor those with palmitoyl CoA as substrate of cyanide-insensitive palmitoyl CoA oxidase support PPARα activation as cause of or contribution to the hepatocarcinogenicity of NVP.

### Gene expression alterations in cultivated rat hepatocytes

**Fig. 5:**
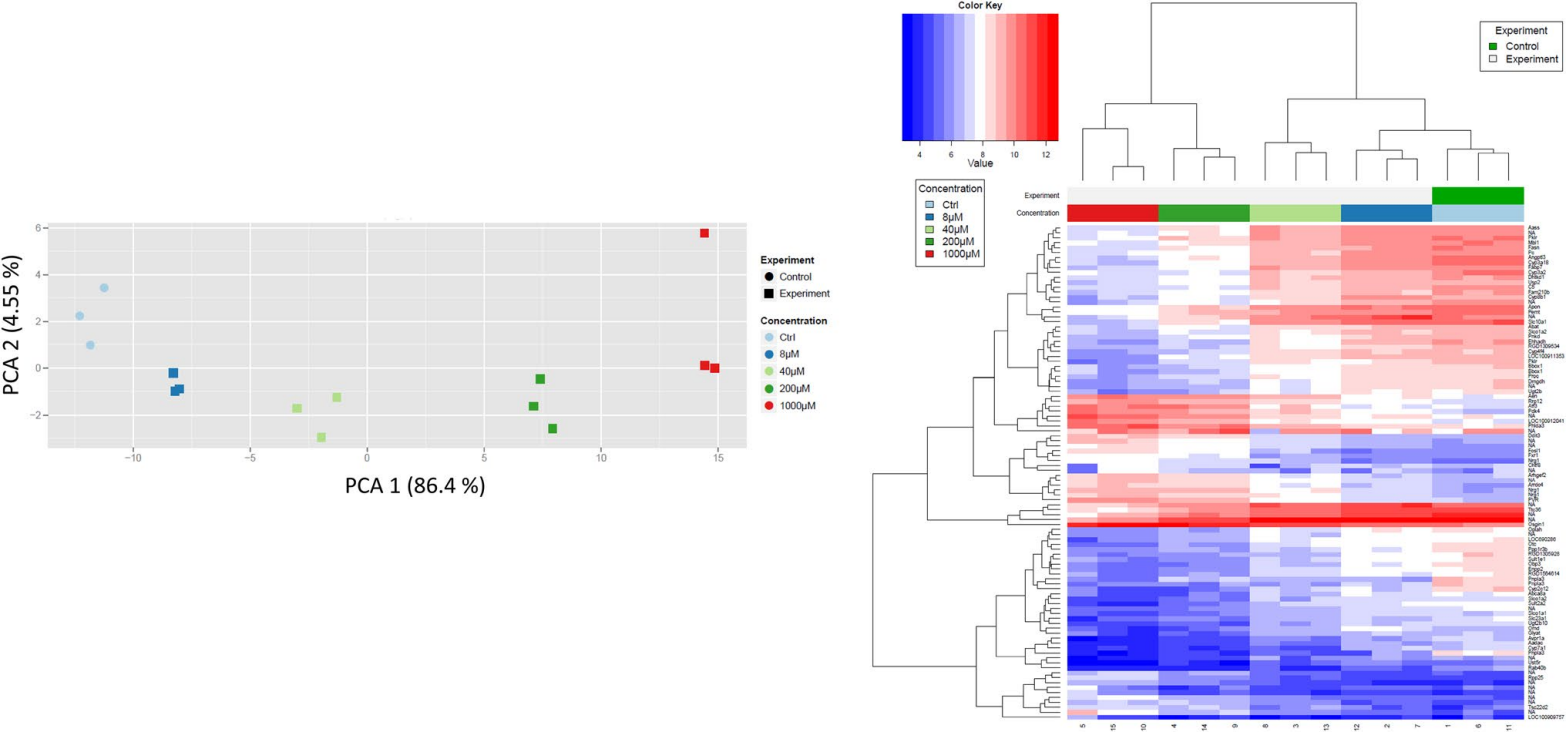
Gene array analysis of cultivated rat hepatocytes after incubation with the indicated concentrations of NVP for 24h.** a** Principle component analysis. PCA: principle component analysis; the percentages in brackets indicate the explained variance of the respective principle component. The three symbols per color indicate the results of three independent incubations.** b** Heatmap visualization of differential alterations of gene expression by NVP. The heatmap is based on the genes with the lowest adjusted p values according to the Limma t test. The colours of the heatmap indicate the relative gene regulation level above (red) or below (blue) the average for each row (color figure online)

To study a possible influence on gene expression, rat hepatocytes were cultivated and incubated with 8, 40, 200 and 1000 µM NVP for 24 h and genome-wide expression analyses were performed. Principle component analysis (PCA) showed expression changes already at the lowest concentration of 8 µM which continued up to the highest tested concentration of 1000 µM (Fig. 5). Genome-wide expression results were visualized in a principal component analysis (Fig. 5a) and the genes with the lowest false discovery rate (fdr) adjusted *p* values were summarized in the heatmap (Fig. 5b). Among the strongest upregulated genes were Pyruvate Dehydrogenase Kinase 4 (PDK4; 8.8-, 5.9- and 3.5-fold at 1000, 200 and 40 µM, respectively; adj. *p* < 0.001), Neuregulin 1 (Nrg1; 7.3-, 6.2- and 3.5-fold at 1000, 200 and 40 µM, respectively; adj. *p* < 0.001) and Activating Transcription Factor 3 (Atf3; 7.1 and 5.1 at 1000 and 200 µM, respectively; adj. *p* < 0.001). Gene ontology (GO) enrichment analysis identified ribosome biogenesis (adj. *p* value: 1 × 10^–18^) and rRNA processing (adj. p-value: 9.9 × 10^–7^) as the strongest enriched motives among upregulated and oxidation–reduction processes (adj. *p* value: 3.4 × 10^–7^) among upregulated genes. Thus, the results show strong gene expression changes at non-cytotoxic NVP concentrations (no cytotoxicity observed in MTT assay up to 1000 µM) (for upregulated and downregulated genes see Supplement 1 and 2, respectively).

## Discussion

Previous studies had shown that NVP was carcinogenic in rats (Klimisch et al. 1997a), but failed to show any genotoxicity. The goal of the present investigation was to investigate with additional genotoxicity experiments whether the lack of NVP-generated genotoxicity would still hold in studies complementing the already existing results and, if so, to investigate further possible mechanisms potentially responsible for the observed carcinogenicity.

Available information reported by ToxCast includes findings that NVP was active in 10 out of 847 in vitro assays. Of interest with respect to our present study are the findings that NVP influences the p53 transcription factor activity and activates the estrogen receptor α, but the ToxCast Pathway models did not render any activity at either the estrogen or the androgen receptors. The AC50 of the p53 transcription factor activity modulation was 0.04 µM, orders of magnitude lower than the highest NVP concentrations used in the genotoxicity tests in the present study, which all remained negative (Ames test about 6 orders of magnitude, chromosome aberration test about 5 orders of magnitude). The p53 transcription factor activity is indicative of the human tumor protein 53 (TP53) DNA binding activity. P53 can be activated by DNA damage, but the TP53 assay does not directly measure genotoxicity. TP53 codes for a tumor suppressor protein containing domains for oligomerization, DNA binding and transcriptional activation. It responds to cellular stress and regulates target genes leading to cell cycle arrest, apoptosis and senescence as well as DNA repair and modulation of metabolism. (https://comptox.epa.gov/dashboard/dsstoxdb/results?search=DTXSID2021440#bioactivity).

In the present study, exposures of rats to 5 ppm, 10 ppm and 20 ppm NVP by inhalation corresponding to the positive carcinogenicity experiments (Klimisch et al. 1997a) led to only mild clinical symptoms such as red nose discharge and increased salivation in some of the exposed animals and to reduced weight or reduced weight increases in the mid- and high-dose groups while in the low-dose group no effects on the weight were observed, all of this similar to the observations in the carcinogenicity study (Klimisch et al. 1997a).

A previous internal study (BASF 1993) had already led to negative results in a micronucleus test. This earlier study had been performed in mice and after oral application of NVP, i.e. both, animal species as well as route of administration differed from the conditions used in the positive carcinogenicity study (Klimisch et al. 1997a). In the present micronucleus test NVP was applied by inhalation to rats, route of exposure and target species (as well as doses) corresponding to the positive carcinogenicity test (Klimisch et al. 1997a). The present micronucleus test in rats was negative confirming the negative results of the previous study in mice, but in the present study under conditions corresponding to the conditions used in the positive carcinogenicity test.

The quotient PCE:NCE, a measure for the toxicity of a test compound for the bone marrow and, hence, a criterion for the successful arrival of a test compound to the bone marrow, was after treatment of the male rats with the high (20 ppm) NVP dose in comparison with the vehicle-treated control reduced by 10%. This may be taken as a sign that NVP successfully arrived in the bone marrow. However, the weakness of the effect precludes a firm conclusion. Yet, previous studies (Klimisch et al. 1997b) already had shown a decrease in the hemoglobin, the hematocrit and the erythrocytes after treatment of rats (two strains: Sprague Dawley and Fischer 344) as well as mice (C57BL/6NCrl) after NVP inhalation starting at 15 ppm and starting at an exposure time of 6 weeks indicating presence and toxicological activity of inhaled NVP in the bone marrow.

Thus, the present micronucleus test was negative under conditions corresponding to those used in the positive carcinogenicity study and under conditions which at least strongly suggest that the test compound successfully arrived at the target cells in the bone marrow [in addition, in the present investigation NVP metabolites were observed in the rat urine, an additional indication for the systemic availability of NVP (details to be published in a future study on the NVP metabolism)].

The Comet assay in the present study also was negative under conditions leading to clear positive results after treatment of the rats with the positive control substance EMS. The Comet assay in the present study was performed after exposure of the rats to the carcinogenic doses of 5 ppm, 10 ppm and 20 ppm NVP by inhalation using the carcinogenicity target organ, the liver, and the organ of first contact, the lung. The Comet assay was performed according to standard protocol as well as in the presence of the DNA repair enzyme FPG.

No cytotoxicity after treatment with NVP was noticed in either of the two organs using dye exclusion test immediately after removing the organs from the body (viability > 90%). In addition, histopathological examination of the liver by hematoxylin–eosin staining showed no increase of necrotic or apoptotic cells after NVP treatment compared with the materials taken from vehicle-treated controls, although these latter results need to be taken with caution since the material from both, NVP-treated as well as vehicle-treated animals had undergone freezing and thawing thereby potentially producing artefacts. A further parameter controversially discussed in the literature as potential marker for cytotoxicity in the Comet assay (pro: Bowen et al., 2011; Henderson et al., 1998; Olive & Banath, 1995; against: Vasquez, 2012; Meintières et al. 2003; Lorenzo et al., 2013; Rundell et al., 2003) is the occurrence of hedgehogs (comets with a very long and diffuse tail and no or a very small head). Guerard et al. (2014) reported that storage of the single-cell suspension at room temperature led to an increase of hedgehogs already after 1 h, but storage on ice up to 8 h led to no increase of hedgehogs. In the present Comet assay neither a dose-dependent increase of the number of hedgehogs was observed after treatment of the rats with NVP nor a significant difference in any of the NVP exposure groups compared with the vehicle-treated controls. This was true for the liver and for the lung in the standard Comet assay and in the assay in presence of FPG. The number of hedgehogs in the EMS-treated positive controls was increased in the liver and in the lung using the standard assay and also in the assay in presence of FPG. The magnitude of this increase was close to that described in the literature for treatment with EMS (Guerard et al., 2014; Stankowski et al., 2015).

The positive controls in the present Comet assay led to increases in the tail intensity for the lung which were two- to threefold lower than historical controls of the testing laboratory which had been obtained with a higher dose of EMS (300 mg/kg body weight) than the dose in the present study (200 mg/kg body weight). No historical control values obtained in the test laboratory were available for the liver, but the values obtained in the present study (17–19%) were close to those described in the literature (14% by O'Donovan and Burlinson, 2013; 20% by Guerard et al., 2014 and by McNamee and Bellier, 2015).

Thus, the negative results in the Comet assay after treatment of the rats with NVP can rightfully be considered as valid in the light of the absence of cytotoxicity under the test conditions as well as the valid positive EMS controls and the vehicle-treated negative controls.

In the present investigations no indications on a DNA damaging potential of NVP were obtained, neither in the liver nor in the lung. The Comet assay detects DNA strand breaks as well as alkali-labile sites. In the modification of the Comet assay by addition of FPG the following additional DNA lesions are transformed into DNA strand breaks and as such become additionally visible: 8-hydroxyguanine, Fapy-guanine (2,4-diamino-6-hydroxy-5-formylaminopyrimidine), Fapy-adenine (2,6-diamino-5-formylamino-pyrimidine) as well as regular and 4’-oxidized abasic sites (Epe et al. 1993). Furthermore, FPG can detect (Azqueta et al., 2013; Speit et al., 2004] and repair (Gill et al., 1996; He et al., 2002; Li et al., 1997) N7-alkylated guanine. Thus, the present results did not provide any evidence that NVP directly induces DNA strand breaks nor akali-labile sites, nor oxidative DNA damage, nor introduction of abasic sites by destabilization of the sugar–phosphate linkage in the DNA, nor a N7-guanine alkylation. Therefore, genotoxicity of NVP connected with any of these mechanisms can be excluded.

As discussed above, an increase in the Comet migration velocity due to exposure to NVP was not observed in any instance. On the contrary, a decrease of the migration was observed after NVP treatment in a few instances, namely in the conventional Comet assay in lung cells of the male rat after treatment of the animals with 20 ppm NVP and in the FPG-modification of the Comet assay in lung cells of the male rat after treatment with 5 ppm and 20 ppm NVP. This may in principle be explained by cross links in the DNA. However, this explanation is improbable, because a DNA binding study in male rats (CD rats) showed no binding of NVP or its metabolites to (liver) DNA after (i.p.) treatment of the rats with ^14^C-NVP (*N*-vinyl[α,ß-^14^C]-2-pyrrolidon und *N*-vinyl-2-pyrrolidone[5-^14^C], 150 and 300 mg/kg body; livers removed 5 h after a single treatment and 1 h after the last of the five consecutive treatments) (Inveresk Research International (IRI) 1986). If NVP or some metabolite derived from it would lead to DNA cross links, one would expect to see binding to DNA. Moreover, the decrease of migration velocity was not seen in the liver, which is a target organ for the NVP-induced carcinogenicity, but only in the lung, which is not a target. Moreover, the decrease in migration was seen in males only, while both genders are equally targeted by the NVP carcinogenicity. Hence, even if putative cross links in the male lung were real, they would not qualify as possible reason for the observed carcinogenicity of NVP.

In summary, the genotoxicity tests on NVP of the present study complement and confirm the already available negative genotoxicity tests. The totality of these genotoxicity tests now comprises the following:

- Several Ames tests in Salmonella typhimurium TA100, TA 98, TA1537 and TA1535 in presence and absence of an exogenous xenobiotica-metabolizing system (S9) from Aroclor-1254-induced rat livers using up to 10 000 µg NVP/plate (Oesch, 1978; Huntington Research Center, 1978; Knaap, 1985; Simmon and Baden, 1980), some of them including the microsomal epoxide hydrolase inhibitor and GSH depletor TCPO (1,1,1-trichoropropene 2,3-oxide) (Oesch, 1978) which increases the sensitivity toward epoxides and toward innumerable electrophilic genotoxic species which are inactivated by GSH. An Ames test using the strains TA1950, TS24, GW19, TA1537, TA1538, TA1952 and mutant hisG46 performed under potential nitrosating conditions (NVP in presence of nitrite at a nitrite/NVP ratio of 1.6:1) (Murphey-Corb et al. 1983).
- A fluctuation test with Klebsiella pneumoniae (point mutations) (Abstract only; no exogenous xenobiotica-metabolizing system) (Knaap et al. 1985).
- Mutagenicity tests in mammalian cells in vitro with and without exogenous xenobiotica-metabolizing system (Litton Bionetics, 1980a; Knaap, 1985).
- UDS (unscheduled DNA synthesis; DNA repair test) in primary rat hepatocytes (Litton Bionetics 1980b).
- Peroral micronucleus test in mice (BASF 1993); micronucleus test after inhalation of NVP by rats (present study).
- Comet assay after inhalation by rats in presence and absence of FPG (present study).
- Test for chromosome aberrations in human lymphocytes (BASF 1987).
- Cell transformation test in BALB/3T3 cells (Litton Bionetics 1980c).
- Sex-linked recessive lethal test in Drosophila melanogaster (Knaap et al. 1985)

Thus, in the vast array of these tests no indication was obtained for genotoxicity as mechanism mediating the observed carcinogenicity of NVP.

The possibility of NVP-induced oxidative stress as potentially underlying its hepato-carcinogenicity was tested by determination of the oxidation-sensitive and at the same time major hepatic antioxidant GSH (Meister and Anderson 1983; Franco and Cidlowski 2009) as well as its oxidized form GSSG, the GSH:GSSG ratio and the percent contribution of GSH to total GSH plus GSSG. None of these parameters was significantly different in the livers of NVP-treated rats compared with vehicle-treated controls.

The comparison of the results for GSH in the present study (4.0–4.5 µmol/g liver) showed that they were well within the quite large range of values reported in the literature for rat liver: 2.59 (Yilmaz et al. 2009), 4.1 (Thomas et al. 1994), approximately 6.5 (Tietze 1969; Davies et al. 1984), 7.9 (Guan et al. 2003) and 8.2 (Giustarini et al. 2011a, b) µmol/g liver. The literature reported values vary not only with the work-up of the investigated samples and with the analytical methods employed, but also biologically depending on the feeding status: Akerboom and Sies (1981) reported 4.4 µmol GSH/ g liver in fasted rats, but 5.5–7.0 µmol GSH/g liver after feeding.

In apparent contrast to the present study (treatment of rats with NVP for 5 days) previous studies of the same laboratory, in which GSH was not specifically determined, but rather as total non-protein-bound sulfhydryls, reported an increase of these total non-protein-bound sulfhydryls (after treatment of Sprague–Dawley rats by inhalation for 3 months with 10 ppm NVP, after 6 months in C57BL mice and F344 rats and after 1 week treatment with 15 ppm NVP) (Klimisch et al. 1997b). As a speculation, a somewhat longer treatment of rats with NVP than the 5 days of the present study may lead to a compensatory increase of non-protein-bound sulfhydryls.

Changes in the concentration of hepatic GSSG may represent a more sensitive parameter as consequence of oxidative stress than those of GSH, since GSSG is present in the liver in much lower concentrations (GSSG concentrations nM in contrast to µM concentrations of GSH). In the present study GSSG concentrations were not significantly different after NVP treatment compared with the vehicle-treated controls. The values were within same magnitude of those reported in the literature: Present study 116–137 nmol GSSG/g liver compared with 18 (Akerboom and Sies, 1981)], 43.3 (Giustarini et al. 2016), 127 (Tietze 1969), 150 (Akerboom and Sies 1981), 190 (Yilmaz et al. 2009), 240 (Davies et al. 1984), 348 (Guan et al. 2003) nmol GSSG/g liver.

In addition, the percent GSH on “total glutathione” (GSH plus GSSG) and the GSH:GSSG ratio in the present study were not significantly different after NVP treatment compared with vehicle-treated controls and were similar to those reported in the literature (Kelly et al. 1998; Owen and Butterfield 2010; Giustarini et al. 2011a, b), as well as within the values calculated from other data in the following publications: Tietze 1969, Davies et al. 1984, Griffith 1980, Guan et al. 2003.

Thus, all parameters of the glutathione status determined in the present studies lay well within the range of literature data and none of them was changed by the treatment with NVP. In addition, the tail intensities in the Comet assay in presence of FPG also were not increased by the treatment with NVP, which represents an additional argument for no increase of oxidative stress. Hence, the present studies did not give any indication that oxidative stress may be the underlying mechanism of or significantly contribute to the observed carcinogenicity of NVP.

Potential activations of hepatic receptors as possible mechanisms underlying the or contributing to the observed hepatocarcinogenicity of NVP also yielded negative results. As a measure of the potential activations of the investigated receptors (aryl hydrocarbon receptor AhR, constitutive androstan receptor CAR, pregnan-X-receptor PXR and peroxisome-proliferator-activated receptor alpha PPARα) enzyme activities were determined which are known to be increased as one of the results of the activation of these receptors (Elcombe et al. 2014; Pelkonen et al. 2008).

Activation of AhR had been shown to be associated with liver tumor promotion. Thus, in a tumor-promotion experiment mice with a constitutively activated AhR developed more liver tumors than wild-type mice (Moennikes et al. 2004). Activation of AhR leads to a very high increase of hepatic CYP1A1 which in turn leads to an accordingly very high increase of EROD activity (Burke et al. 1994). In the present experiments no significant change of the hepatic EROD activity was seen after treatment of rats with NVP at doses corresponding to those which had shown the hepatocarcinogenicity of NVP (Klimisch et al. 1997a). The hepatic EROD activities observed in the present study (17–22 pmol/min/mg protein) were within the activities reported in the literature (47 ± 39 pmol/min/mg protein (Tabrez and Ahmad 2010; Wardlaw et al. 1998). Thus, no indication for AhR activation as mechanism or contributing factor for the carcinogenicity of NVP was obtained.

A potential activation of CAR by NVP is with respect to a potential mechanism of its carcinogenicity of special interest since CAR activation had been shown to lead to an increase of liver cell proliferation (Elcombe et al. 2014). Previous studies in our laboratory had shown that NVP exposure leads to liver cell proliferation already at quite low doses (BASF 2011): Treatment of rats by inhalation for 28 days with 0.5 ppm NVP led to a significant increase of proliferation in zones 3 and 2 (centrilobular and midzonal), which in zone 3 clearly dose-dependently increased at higher doses. This localization of the NVP-stimulated proliferation increase is of special interest, since the increase of CYP2B by the CAR-activator phenobarbital (Negishi et al. 2020) is localized in zones 3 and 2, preferentially in zone 3 (Wolf et al. 1984). Treatment of rats for a shorter time period, 7 days, led to a significant increase starting at 1 ppm NVP. Apoptosis was also observed but only at doses higher than those needed for an increase of proliferation and with a different distribution within the liver lobules indicating that the NVP-induced liver cell proliferation was mitogenic rather than the result of a toxic insult of NVP to the liver. This conclusion is further supported by results from subchronic studies in rats and mice in which necrosis of single centrilobular liver cells occurred only at considerably higher doses (45 ppm) of NVP (Klimisch et al. 1997b).

Activation of CAR and PXR leads to increases of hepatic CYP2B and CYP3A, CAR preferentially to increases of CYP2B, PXR preferentially to increases of CYP3A (Tolson and Wang 2010; Wang et al. 2012) and, accordingly, to preferential increases of PROD and BROD activities, respectively. In the present study no significant increase of liver or lung PROD or BROD activities upon treatment of rats with NVP was observed, while the classical CYP2B inducer phenobarbital led to reported massive increases of hepatic PROD activities by 50 to 140-fold (Waxmann 1999; Lubet et al. 1985; Tompkins and Wallac 2007). Masking of potential NVP-mediated increases of PROD activities by NVP metabolites as shown for cyclic tertiary amines (Hollenberg et al. 2008) is highly improbable since co-incubation of 1, 10 or 100 µM NVP did not inhibit PROD activity in the present study. Thus, no indication for CAR activation as mechanism or contributing factor for the carcinogenicity of NVP was obtained in the present study.

A further potential non-genotoxic mechanism of the NVP-induced hepatocarcinogenesis could conceivably be an activation of PPARα which induces peroxisome proliferation, liver cell proliferation and hepatocarcinogenesis in rodents [albeit not relevant for humans (Gonzales et al. 1998; Gonzales and Shah 2008; Johnson et al. 2002; Corton et al. 2014; Kim et al. 2013)].

Activation of PPARα leads to induction of CYP4A and of the cyanide-insensitive palmitoyl-CoA-oxidase/acyl-CoA-oxidase (Kim et al. 2013; Klaunig et al. 2003; Suga 2004). The former is catalyzing the ω-hydroxylation of fatty acids which subsequently are further oxidized to dicarbonic acids used in the peroxisomal fatty acid ß-oxidation, the latter is the first enzyme in the sequence of peroxisomal fatty acid ß-oxidation (Reddy and Hashimoto 2001; Reubsaet et al. 1988; Yelandi et al. 2000).

In the present study a slight (1.3-fold), but not statistically significant, increase of the CYP4A-mediated fatty acid ω-hydroxylation and no increase of the cyanide-insensitive palmitoyl-CoA-oxidase/acyl-CoA-oxidase were observed, while in the literature after treatment with peroxisome proliferators high (up to 13-fold) increases of the former (Nilsson et al. 1986; Suga 2004) and very massive (up to more than 300-fold) of the latter (Gonzales et al. 1998; Klaunig et al. 2003; Suga, 2004) were described.

In addition, it had been described that activation of PPARα led to induction of oxidative stress due to the increase of fatty acid metabolism leading to an increase of reactive oxygen species, especially an increase of the cyanide-insensitive palmitoyl-CoA-oxidase/acyl-CoA-oxidase leading to hydrogen peroxide as secondary product of the enzymatic reaction (Klaunig et al. 2003; Melnick et al. 1996; Misra and Reddy 2014; Yeldani et al. 2000). However, as already discussed no increase of oxidative stress was seen in the livers of rats after treatment with NVP. Thus, no indication for PPARα activation as mechanism or contributing factor for the carcinogenicity of NVP was obtained in the present study.

A feature of many non-genotoxic carcinogens is that they induce gene expression changes at non cytotoxic concentrations. To address this aspect, we studied cultivated rat hepatocytes by microarrays. Cultivated hepatocytes were used because they allow the analysis of concentration ranges for which cytotoxic effects can be excluded, which would be more challenging in the in vivo situation. The results clearly show that non-cytotoxic concentrations as low as 8 and 40 µM caused relatively strong expression changes (no cytotoxicity observed up to 1000 µM in the MTT assay). Among the most upregulated genes were PDK4 which is known to influence glucose metabolism, the epidermal growth factor receptor ligand NRG1 and the stress response factor ATF3 that depending on cell type and microenvironment have been reported to be involved in carcinogenesis but also in tumor suppression (Chen et al., 2018; Choiniere et al., 2017; Shi et al., 2018). If these gene expression alterations play a critical role in NVP induced non-genotoxic carcinogenesis remains to be studied.

In summary, based on the overall available data and on the investigations within the current study, NVP can be assessed to be a non-genotoxic carcinogen. The knowledge on mode of action (MoA) for carcinogenicity is essential for risk assessors, since the first decision when developing a risk assessment is the determination of the likely MoA and whether the critical effect observed is threshold-based or not. For stochastic types of toxicity, especially mutagenicity and genotoxic carcinogenicity, the default assumption prevailing in current regulatory schemes is that there is no threshold and the dose–response relation is based, in principle, on linear extrapolation to a dose of ‘very low concern’. In the case of NVP, there is sufficient evidence from experimental data to justify a threshold-based approach for risk assessment.

## References

[CR1] Akerboom TPM, Sies H (1981). Assay of glutathione disulfide and glutathione mixed disulfides in biological samples. Methods Enzymol.

[CR2] Azqueta A, Arbillaga L, Lopez de Cerain A, Collins A (2013). Enhancing the sensitivity of the comet assay as a genotoxicity test, by combining it with bacterial repair enzyme FPG. Mutagenesis.

[CR3] BASF (1987) In vitro cytogenetic investigation of *N*-vinylpyrrolidone-2 in human lymphocytes. Unpublished result. Report No. 0161/8616 Ludwigshafen am Rhein, Germany

[CR4] BASF AG (1993) Cytogenetic Study In Vivo of *N*-Vinylpyrrolidone-2 (NVP) in Mice Micronucleus Test Single Oral Administration. BASF AG, Department of Toxicology, unpublished

[CR5] BASF SE (2011) *N*-Vinyl-2-pyrrolidone, subacute 28 day inhalation study in Wistar rats, vapor exposure. 40I0572/07038, BASF SE, Department of Toxicology, upublisehed

[CR6] Bowen DE, Whitwell JH, Lillford L, Henderson D, Kidd D, McGarry S, Pearce G, Beevers C, Kirkland DJ (2011). Evaluation of a multi-endpoint assay in rats, combining the bone-marrow micronucleus test, the Comet assay and the flow-cytometric peripheral blood micronucleus test. Mutat Res.

[CR7] Bradford MM (1976). A rapid and sensitive method for the quantitation of microgram quantities of protein utilizing the principle of protein-dye binding. Anal Biochem.

[CR8] Burke MD, Thompson S, Weaver RJ, Wolf CR, Mayer RT (1994). Cytochrome P450 specificities of alkoxyresorufin O-dealkylation in human and rat liver. Biochem Pharmacol.

[CR9] Chen C, Chao G, Zheng L, Liangyu L, Fangyu Z, Hua T, Taoyang C, Hong L, Ming Y, Jinjun L (2018). ATF3 inhibits the tumorigenesis and progression of hepatocellular carcinoma cells via upregulation of CYR61 expression. J Exp Clin Cancer Res.

[CR10] Choiniere J, Wu J, Wang L (2017). Pyruvate dehydrogenase kinase 4 deficiency results in expedited cellular proliferation through E2F1-mediated increase of cyclins. Mol Pharmacol.

[CR11] Collins AR, Oscoz AA, Brunborg G, Gaivao I, Giovannelli L, Kruszewski M, Smith CC, Stetina R (2008). The comet assay: topical issues. Mutagenesis.

[CR12] Corton JC, Cunningham ML, Hummer BT, Lau C, Meek B, Peters JM, Popp JA, Rhomberg L, Seed J, Klaunig JE (2014). Mode of action framework analysis for receptor-mediated toxicity: The peroxisome proliferator-activated receptor alpha (PPARalpha) as a case study. Crit Rev Toxicol.

[CR13] Davies MH, Birt DF, Schnell RC (1984). Direct enzymatic assay for reduced and oxidized glutathione. J Pharmacol Methods.

[CR14] Elcombe CR, Peffer RC, Wolf DC, Bailey J, Bars R, Bell D, Cattley RC, Ferguson SS, Geter D, Goetz A, Goodman JI, Hester S, Jacobs A, Omiecinski CJ, Schoeny R, Xie W, Lake BG (2014). Mode of action and human relevance analysis for nuclear receptor-mediated liver toxicity: a case study with phenobarbital as a model constitutive androstane receptor (CAR) activator. Crit Rev Toxicol.

[CR15] Epe B, Pflaum M, Häring M, Hegler J, Rüdiger H (1993). Use of repair endonucleases to characterize DNA damage induced by reactive oxygen species in cellular and cell-free systems. Toxicol Lett.

[CR16] Franco R, Cidlowski JA (2009). Apoptosis and glutathione: beyond an antioxidant. Cell Death Differ.

[CR17] Gallagher EP, Kavanagh TJ, Eaton DL (1994). Glutathione oxidized glutathione, and mixed disulfides in biological samples. Methods Toxicol.

[CR18] Gill RD, Cussac C, Souhami RL, Laval F (1996). Increased resistance to *N*, *N*′, *N*″-triethylenethiophosphoramide (thiotepa) in cells expressing the *Escherichia coli* formamidopyrimidine-DNA glycosylase. Can Res.

[CR19] Giustarini D, Dalle-Donne I, Milzani A, Rossi R (2011). Low molecular mass thiols, disulfides and protein mixed disulfides in rat tissues: influence of sample manipulation, oxidative stress and ageing. Mech Ageing Dev.

[CR20] Giustarini D, Dalle-Donne I, Milzani A, Fanti P, Rossi R (2011). Analysis of GSH and GSSG after derivatization with *N*-ethylmaleimide. Nat Protoc.

[CR21] Giustarini D, Tsikas D, Colombo G, Milzani A, Dalle-Donne I, Fanti P, Rossi R (2016). Pitfalls in the analysis of the physiological antioxidant glutathione (GSH) and its disulfide (GSSG) in biological samples: an elephant in the room. J Chromatogr B Anal Technol Biomed Life Sci.

[CR22] Godoy P, Hewitt NJ, Albrecht U (2013). (2013) Recent advances in 2D and 3D in vitro systems using primary hepatocytes, alternative hepatocyte sources and non-parenchymal liver cells and their use in investigating mechanisms of hepatotoxicity, cell signaling and ADME. Arch Toxicol.

[CR23] Gonzales FJ, Shah YM (2008). PPARalpha: mechanism of species differences and hepatocarcinogenesis of peroxisome proliferators. Toxicology.

[CR24] Gonzales FJ, Jeffrey MP, Cattley RC (1998). Mechanism of action of the nongenotoxic peroxisome proliferators: role of the peroxisome proliferator-activated receptor alpha. J Natl Cancer Inst.

[CR25] Gornall AG, Bardawill CJ, David MM (1949). Determination of serum proteins by means of the biuret reaction. J Biol Chem.

[CR26] Gottwald W (2000). Statistik für Anwender.

[CR97] Griffith OW (1980). Determination of Glutathione and Glutathione Disulfide using Glutathione reductase and 2-Vinylpyridine. Anal Biochem.

[CR27] Grinberg M, Stöber RM, Albrecht W, Edlund K, Schug M, Godoy P, Cadenas C, Marchan R, Lampen A, Braeuning A, Buhrke T, Leist M, Oberemm A, Hellwig B, Kamp H, Gardner I, Escher S, Taboureau O, Aguayo-Orozco A, Sachinidis A, Ellinger-Ziegelbauer H, Rahnenführer J, Hengstler JG (2018). Toxicogenomics directory of rat hepatotoxicants in vivo and in cultivated hepatocytes. Arch Toxicol.

[CR28] Guan X, Hoffman B, Dwivedi C, Matthees DP (2003). A simultaneous liquid chromatography/mass spectrometric assay of glutathione, cysteine, homocysteine and their disulfides in biological samples. J Pharm Biomed Anal.

[CR29] Guerard M, Marchand C, Plappert-Helbig U (2014). Influence of experimental conditions on data variability in the liver comet assay. Environ Mol Mutagen.

[CR30] Hartmann A, Agurell E, Beevers C, Brendler-Schwaab S, Burlinson B, Clay P, Collins A, Smith A, Speit G, Thybaud V, Tice RR (2003). Recommendations for conducting the in vivo alkaline Comet assay. Mutagenesis.

[CR31] Hartmann A, Schumacher M, Plappert-Helbig U, Lowe P, Suter W, Mueller L (2004). Use of the alkaline in vivo Comet assay for mechanistic genotoxicity investigations. Mutagenesis.

[CR32] He YH, Xu Y, Kobune M, Wu M, Kelley MR, Martin WJ (2002). Escherichia coli FPG and human OGG1 reduce DNA damage and cytotoxicity by BCNU in human lung cells. Am J Physiol Lung Cell Mol Physiol.

[CR33] Heise T, Schug M, Storm D, Ellinger-Ziegelbauer H, Ahr HJ, Hellwig B, Rahnenfuhrer J, Ghallab A, Guenther G, Sisnaiske J, Reif R, Godoy P, Mielke H, Gundert-Remy U, Lampen A, Oberemm A, Hengstler JG (2012). In vitro–in vivo correlation of gene expression alterations induced by liver carcinogens. Curr Med Chem.

[CR34] Henderson L, Wolfreys A, Fedyk J, Bourner C, Windebank S (1998). The ability of the comet assay to discriminate between genotoxins and cytotoxins. Mutagenesis.

[CR98] Hollenberg PF, Kent UM, Bumpus NN (2008). Mechanism-based inactivation of human cytochromes p450s: experimental characterization, reactive intermediates, and clinical implications. Chem Res Toxicol.

[CR35] Huntingdon Research Centre (HRC) (1978) Ames metabolic activation test to assess the potential mutagenic effect of *N*-vinyl pyrrolidone. Huntingdon Research Center, unpublished

[CR36] Inveresk Research International (IRI) (1986) *N*-vinyl-2-pyrrolidone: association of 14 C-labelled chemical with rat liver DNA, Inveresk Research International (Sponsor BASF AG), unveröffentlicht

[CR37] JaCVAM (2009) International Validation of the In Vivo Rodent Alkaline Comet Assay for the Detection of Genotoxic Carcinogens. Version 14.2

[CR38] Johnson EF, Hsu MH, Savas U, Griffin KJ (2002). Regulation of P450 4A expression by peroxisome proliferator activated receptors. Toxicology.

[CR39] Kelly KA, Havrilla CM, Brady TC, Abramo KH, Levin ED (1998). Oxidative stress in toxicology: established mammalian and emerging piscine model systems. Environ Health Perspect.

[CR40] Kim S, Kiyosawa N, Burgoon LD, Chang CC, Zacharewski TR (2013). PPARa-mediated responses in human adult liver stem cells: in vivo/in vitro and cross-species comparisons. J Steroid Biochem Mol Biol.

[CR41] Klaunig JE, Babich MA, Baetcke KP, Cook JC, Corton JC, David RM, DeLuca JG, Lai DY, McKee RH, Peters JM, Roberts RA, Fenner-Crisp PA (2003). PPARalpha agonist-induced rodent tumors: modes of action and human relevance. Crit Rev Toxicol.

[CR42] Klimisch HJ, Deckardt K, Gembardt C, Hildebrand B, Kuettler K, Roe FJC (1997). Long-term inhalation toxicity of *N*-vinylpyrrolidone-2 vapours. Studies in rats. Food Chem Toxicol.

[CR43] Klimisch HJ, Deckardt K, Gembardt C, Hildebrand B, Kuettler K, Roe FJC (1997). Subchronic inhalation and oral toxicity of N-vinylpyrrolidone-2. Studies in rodents. Food Chem Toxicol.

[CR44] Knaap AGA, Voogd CE, Kramers PGN (1985). Mutagenicity of vinyl compounds. Mutat Res.

[CR45] Lazarow PB (1981). Assay of peroxisomal ω-oxidation of fatty acids. Methods Enzymol.

[CR46] Li Q, Laval J, Ludlum DB (1997). Fpg protein releases a ringopened N-7 guanine adduct from DNA that has been modified by sulfur mustard. Carcinogenesis.

[CR47] Lillie RD, Ashburn LL (1943). Supersaturated solutions of fat stains in dilute isopropanol for demonstration of acute fatty degeneration not shown by Herxheimer's technique. Arch Pathol.

[CR48] Litton Bionetics (1980a) Mutagenicity Evaluation of V-Pyrol (*N*-Vinyl-2-Pyrrolidone) in the mouse lymphoma forward mutation assay. Litton Bionet unpublished

[CR49] Litton Bionetics (1980b) (for GAF Inc., Wayne, N. Y) Unpublished report: Evaluation of V-Pyrol® (*N*-Vinyl-2-pyrrolidone) in the primary rat hepatocyte unscheduled DNA synthesis assay, Genetics Assay No. 4899, LBI Proj. No. 20991

[CR50] Litton Bionetics (1980c) (for GAF Inc., Wayne, N. Y) Unpublished report: evaluation of V-Pyrol® (*N*-vinyl-2-pyrrolidone) in the in vitro transformation of BALB/3T3 cells assay, Genetics Assay No. 4899, LBI Proj. No. 20992

[CR51] Lorenzo Y, Costa S, Collins AR, Azqueta A (2013). The comet assay, DNA damage, DNA repair and cytotoxicity: hedgehogs are not always dead. Mutagenesis.

[CR52] Lovell DP, Omori T (2008). Statistical issues in the use of the comet assay. Mutagenesis.

[CR53] Lubet RA, Mayer RT, Cameron JW, Nims RW, Burke MD, Wolff T, Guengerich FP (1985). Dealkylation of pentoxyresorufin: a rapid and sensitive assay for measuring induction of cytochrome(s) P-450 by phenobarbital and other xenobiotics in the rat. Arch Biochem Biophys.

[CR54] MAK (1994) Gesundheitsschädliche Arbeitsstoffe; Toxikologisch-arbeitsmedizinische Begründung von MAK-Werten. 1–20. Lieferung 1994, pp. N1–14. VCH­Verlagsgesellschaft, Weinheim

[CR55] McNamee JP, Bellier PV (2015). Use of a standardized JaCVAM in vivo rat comet assay protocol to assess the genotoxicity of three coded test compounds; ampicillin trihydrate, 1,2-dimethylhydrazine dihydrochloride, and *N*-nitrosodimethylamine. Mutat Res Gen Toxicol Environ Mutagen.

[CR56] Meintières S, Nesslany F, Pallardy M, Marzin D (2003). Detection of ghost cells in the standard alkaline comet assay is not a good measure of apoptosis. Environ Mol Mutagen.

[CR57] Meister A, Anderson ME (1983). Glutathione. Annu Rev Biochem.

[CR58] Melnick RL, Kohn MC, Portier CJ (1996). Implications for risk assessment of suggested nongenotoxic mechanisms of chemical carcinogenesis. Environ Health Perspect.

[CR59] Misra P, Reddy JK (2014). Peroxisome proliferator-activated receptor-alpha activation and excess energy burning in hepatocarcinogenesis. Biochimie.

[CR60] Moennikes O, Loeppen S, Buchmann A, Andersson P, Ittrich C, Poellinger L, Schwarz M (2004). A constitutively active dioxin/aryl hydrocarbon receptor promotes hepatocarcinogenesis in mice. Can Res.

[CR61] Murphey-Corb M, Kong HL, Murray ML (1983). Mutagenic activity from nitrosation of oligoamines. Environ Mutagen.

[CR62] Nakajima M, Masumori S, Tanaka J, Hayashi M, Uno Y, Kojima H, Tice RR (2009) An atlas of comet images: JaCVAM initiative international validation trial for the in vivo Comet Assay. In: 8th International Comet Assay Workshop

[CR63] Negishi M, Kobayashi K, Sakuma T, Sueyoshi T (2020). Nuclear receptor phosphorylation in xenobiotic signal transduction. J Biol Chem.

[CR64] Nilsson A, Arey H, Pedersen JI, Christiansen EN (1986). The effect of high-fat diets on microsomal lauric acid hydroxylation in rat liver. Biochem Biophys Acta.

[CR65] O'Donovan M, Burlinson B (2013). Maximum dose levels for the rodent comet assay to examine damage at the site of contact or to the gastrointestinal tract. Mutagenesis.

[CR66] Oesch F (1978) (for BASF, D-6700 Ludwigshafen): Ames test for Vinylpyrrolidone, unpublished BASF report, Tox.-No. 77/241, 30. 5. 1978

[CR67] Oesch-Bartlomowicz B, Oesch F (2007) Mechanisms of toxication and detoxication which challenge drug candidates and drugs. In: Comprehensive Medicinal Chemistry (eds. Taylor JB and Triggle DJ), Elsevier, Amsterdam, pp 193–214

[CR68] Olive PL, Banath JP (1995). Sizing highly fragmented DNA in individual apoptotic cells using the comet assay and a DNA crosslinking agent. Exp Cell Res.

[CR69] Owen JB, Butterfield DA (2010). Measurement of oxidized/reduced glutathione ratio. Methods Mol Biol.

[CR70] Pelkonen O, Turpeinen M, Hakkola J, Honkakoski P, Hukkanen J, Raunio H (2008). Inhibition and induction of human cytochrome P450 enzymes: current status. Arch Toxicol.

[CR71] Reddy JK, Hashimoto T (2001). Peroxisomal beta-oxidation and peroxisome proliferator-activated receptor alpha: an adaptive metabolic system. Annu Rev Nutr.

[CR72] Reubsaet FA, Veerkamp JH, Bukkens SG, Trijbels JM, Monnens LA (1988). Acyl-CoA oxidase activity and peroxisomal fatty acid oxidation in rat tissues. Biochimica Biophysica Acta.

[CR73] Romagna F, Staniforth CD (1989). The automated bone marrow micronucleus test. Mutat Res.

[CR74] Romeis,  (2010). Mikroskopische Technik.

[CR75] Rundell MS, Wagner ED, Plewa MJ (2003). The comet assay: genotoxic damage or nuclear fragmentation?. Environ Mol Mutagen.

[CR76] Salamone M, Heddle JA, Stuart E, Katz M (1980). Towards an improved micronucleus test: studies on 3 model agents, mitomycin C, cyclophosphamide and dimethylbenzanthracene. Mutat Res.

[CR77] Shi DM, Li-Xin L, Xin-Yu B, Xue-Jiang S, Li-Li L, Hong-Xin Z, Ting-Jia P, Jian Z, Jia F, Wei-Zhong Wu (2018). miR-296-5p suppresses EMT of hepatocellular carcinoma via attenuating NRG1/ERBB2/ERBB3 signaling. J Exp Clin Cancer Res.

[CR78] Simmon VF, Baden JM (1980). Mutagenic activity of vinyl compounds and derived epoxides. Mutat Res.

[CR79] Singh NP, McCoy MT, Tice RR, Schneider EL (1988). A simple technique for quantitation of low levels of DNA damage in individual cells. Exp Cell Res.

[CR80] Speit G, Schuetz P, Bonzheim I, Trenz K, Hoffmann H (2004). Sensitivity of the FPG protein towards alkylation damage in the comet assay. Toxicol Lett.

[CR81] Stankowski LF, Aardema MJ, Lawlor T, Pant K, Roy S, Xu Y, Elbekai R (2015). Integration of Pig-a, micronucleus, chromosome aberration and comet assay endpoints in a 28-day rodent toxicity study with urethane. Mutagenesis.

[CR82] Strober W (2001). Trypan blue exclusion test of cell viability. Current protocols in immunology.

[CR83] Suga T (2004). Hepatocarcinogenesis by peroxisome proliferators. J Toxcicol Sci.

[CR84] Tabrez S, Ahmad M (2010). Cytochrome P450 system as a toxicity biomarker of industrial wastewater in rat tissues. Food Chem Toxicol.

[CR85] Thomas H, Strolin-Benedetti M, Dostert P, Oesch F (1994). The effect of indobufen on the activities of selected rat liver phase I and phase II drug metabolizing enzymes, peroxisomal beta-oxidation and hepatic glutathione status. J Pharm Pharmacol.

[CR86] Tice RR, Agurell E, Anderson D, Burlinson B, Hartmann A, Kobayashi H, Miyamae Y, Rojas E, Ryu JC, Sasaki YF (2000). Single cell gel/comet assay: guidelines for in vitro and in vivo genetic toxicology testing. Environ Mol Mutagen.

[CR87] Tietze F (1969). Enzymatic method for quantitative determination of nanogram amounts of total and oxidized glutathione: applications to mammalian blood and other tissues. Anal Biochem.

[CR88] Tolson AH, Wang H (2010). Regulation of drug-metabolizing enzymes by xenobiotic receptors: PXR and CAR. Adv Drug Deliv Rev.

[CR89] Tompkins LM, Wallace AD (2007). Mechansims of cytochrome P450 induction. J Biochem Mol Toxicol.

[CR90] Vasquez MZ (2012). Recommendations for safety testing with the in vivo comet assay. Mutat Res Gen Toxicol Environ Mutagen.

[CR91] Wang YM, Ong SS, Chai SC, Chen T (2012). Role of CAR and PXR in xenobiotic sensing and metabolism. Expert Opin Drug Metab Toxicol.

[CR92] Wardlaw SA, Nikula KJ, Kracko DA, Finch GL, Thornton-Manning JR, Dahl AR (1998). Effect of cigarette smoke on CYP1A1, CYP1A2 and CYP2B1/2 of nasal mucosae in F344 rats. Carcinogenesis.

[CR93] Waxman DJ (1999). P450 gene induction by structurally diverse xenochemicals: central role of nuclear receptors CAR, PXR, and PPAR. Arch Biochem Biophys.

[CR94] Wolf CR, Moll E, Friedberg T, Oesch F, Buchmann A, Kuhlmann WD, Kunz HW (1984). Characterization, localization and regulation of a novel phenobarbital-inducible form of cytochrome P450, compared with three further cyt. P450-isoenzymes, NADPH cyt. P450-reductase, glutathione transferases and microsomal epoxide hydrolase. Carcinogenesis.

[CR95] Yeldandi AV, Rao MS, Reddy JK (2000). Hydrogen peroxide generation in peroxisome proliferator-induced oncogenesis. Mutat Res.

[CR96] Yilmaz Ö, Keser S, Tuzcu M, Güvenc M, Cetintas B, Irtegün S, Tastan H, Sahin K (2009). A practical method to measure reduced (GSH) and oxidized (GSSG) glutathione concentrations in animal tissues. J Anim Vet Adv.

